# Self-Organization of the *Escherichia coli* Chemotaxis Network Imaged with Super-Resolution Light Microscopy

**DOI:** 10.1371/journal.pbio.1000137

**Published:** 2009-06-23

**Authors:** Derek Greenfield, Ann L. McEvoy, Hari Shroff, Gavin E. Crooks, Ned S. Wingreen, Eric Betzig, Jan Liphardt

**Affiliations:** 1 Biophysics Graduate Group, University of California Berkeley, Berkeley, California, United States of America; 2 Physical Biosciences Division, Lawrence Berkeley National Laboratory, Berkeley, California, United States of America; 3 Howard Hughes Medical Institute, Janelia Farm Research Campus, Ashburn, Virginia, United States of America; 4 Department of Molecular Biology, Princeton University, Princeton, New Jersey, United States of America; 5 Department of Physics, University of California Berkeley, Berkeley, California, United States of America; Harvard University, United States of America

## Abstract

The *Escherichia coli* chemotaxis network is a model system for biological signal processing. In *E. coli*, transmembrane receptors responsible for signal transduction assemble into large clusters containing several thousand proteins. These sensory clusters have been observed at cell poles and future division sites. Despite extensive study, it remains unclear how chemotaxis clusters form, what controls cluster size and density, and how the cellular location of clusters is robustly maintained in growing and dividing cells. Here, we use photoactivated localization microscopy (PALM) to map the cellular locations of three proteins central to bacterial chemotaxis (the Tar receptor, CheY, and CheW) with a precision of 15 nm. We find that cluster sizes are approximately exponentially distributed, with no characteristic cluster size. One-third of Tar receptors are part of smaller lateral clusters and not of the large polar clusters. Analysis of the relative cellular locations of 1.1 million individual proteins (from 326 cells) suggests that clusters form via stochastic self-assembly. The super-resolution PALM maps of *E. coli* receptors support the notion that stochastic self-assembly can create and maintain approximately periodic structures in biological membranes, without direct cytoskeletal involvement or active transport.

## Abstract 2

Photoactivated localization microscopy analysis of chemotaxis receptors in bacteria suggests that the non-random organization of these proteins results from random self-assembly of clusters without direct cytoskeletal involvement or active transport.

## Author Summary

Cells arrange their components—proteins, lipids, and nucleic acids—in organized and reproducible ways to optimize the activities of these components and, therefore, to improve cell efficiency and survival. Eukaryotic cells have a complex arrangement of subcellular structures such as membrane-bound organelles and cytoskeletal transport systems. However, subcellular organization is also important in prokaryotic cells, including rod-shaped bacteria such as *E. coli*, most of which lack such well-developed systems of organelles and motor proteins for transporting cellular cargoes. In fact, it has remained somewhat mysterious how bacteria are able to organize and spatially segregate their interiors. The *E. coli* chemotaxis network, a system important for the bacterial response to environmental cues, is one of the best-understood biological signal transduction pathways and serves as a useful model for studying bacterial spatial organization because its components display a nonrandom, periodic distribution in mature cells. Chemotaxis receptors aggregate and cluster into large sensory complexes that localize to the poles of bacteria. To understand how these clusters form and what controls their size and density, we use ultrahigh-resolution light microscopy, called photoactivated localization microscopy (PALM), to visualize individual chemoreceptors in single *E. coli* cells. From these high-resolution images, we determined that receptors are not actively distributed or attached to specific locations in cells. Instead, we show that random receptor diffusion and receptor–receptor interactions are sufficient to generate the observed complex, ordered pattern. This simple mechanism, termed stochastic self-assembly, may prove to be widespread in both prokaryotic and eukaryotic cells.

## Introduction

**Figure 1:**
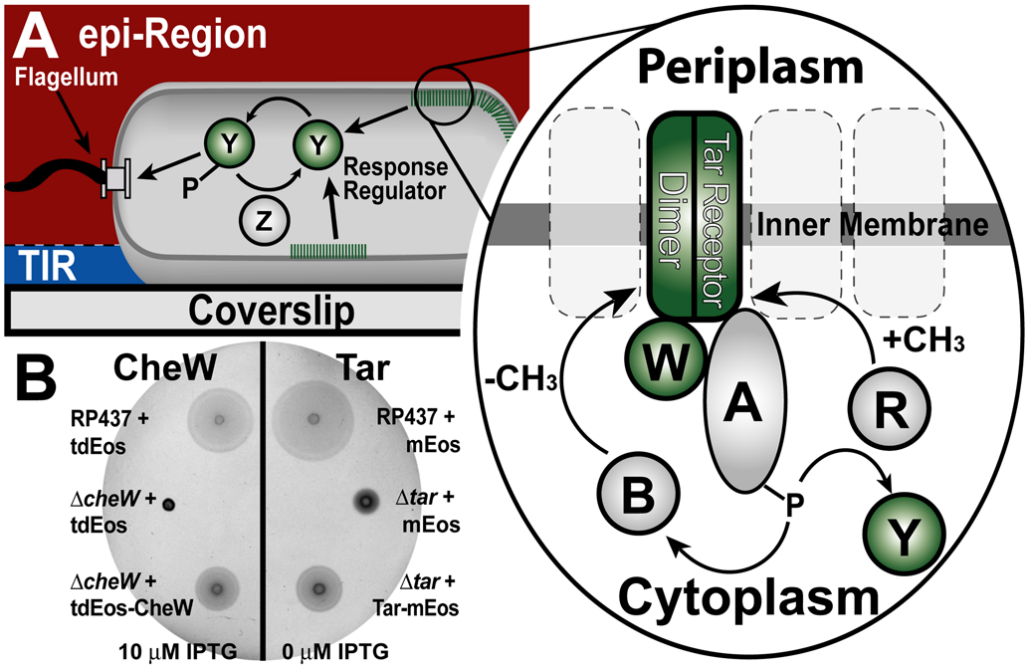
Membrane receptor clusters transduce chemotatic signals. (A) Schematic of *E. coli* cell imaged in PALM. Regions of the cell PALMed in TIR and epi-illumination are shown. Right: zoom of circled region denoted in (A) shows the chemotaxis signal transduction pathway. Proteins in green were labeled with Eos including a receptor dimer (Tar), CheW, and CheY. P denotes phosphate group and CH_3_ is a methyl group. (B) Swarm plates show Eos-tagged chemotaxis proteins support chemotaxis. *E. coli* cells were spotted on minimal phosphate soft-agar plates with 100 µM aspartate and ampicillin, and allowed to swarm for 16–18 h at 30°C (Materials and Methods). Shown are wild-type RP437 cells containing only cytoplasmic Eos (positive control; top), knockout strains with cytoplasmic Eos (negative control; middle), and knockout strains complemented with Eos-tagged chemotaxis proteins (imaged cells; bottom). Complementation demonstrates that Eos-tagged proteins are partially functional, although not as efficient as the wild-type proteins. CheW (left) and Tar (right) fusion proteins support chemotaxis at 10 µM IPTG induction and no induction, respectively (Figure S1). Note that RP437 Δ*tar* cells are weakly chemotactic due to the presence of other receptors.

Efficient biological signal processing often requires complex spatial organization of the signaling machinery. Understanding how this spatial organization is generated, maintained, and repaired inside cells is a fundamental theme of biology. A well-understood signaling network with complex spatial organization is the bacterial chemotaxis system, which directs the movement of cells towards or away from sugars, amino acids, and many other soluble molecules [1]. In *Escherichia coli*, five types of transmembrane chemoreceptors form trimers of dimers [2],[3], which cluster into large complexes containing tens of thousands of proteins [4]–[7]. Receptor clustering enables cooperative interactions between receptors [8]–[11], contributing to a bacterium's ability to sense nanomolar concentrations of chemicals and small fractional changes in chemical concentrations over a wide range [12]–[14]. Chemotaxis clusters are stabilized by the adaptor protein CheW and the histidine kinase CheA, which bind receptors in a ternary complex. CheA transduces signals from membrane receptors to the cytoplasmic response regulator CheY, which diffuses to flagellar motors and modulates their direction of rotation (Figure 1A; for review see [5]).

A variety of imaging studies have advanced our understanding of how the spatial organization of the chemotaxis network arises and contributes to function [15]. Time-lapse fluorescence microscopy suggests that receptors are inserted randomly into the lateral membrane via the general protein translocation machinery and then diffuse to existing clusters [16]. Immunoelectron and fluorescence microscopy have shown that receptor clusters are found at the cell poles [4] and future division sites [17].

Despite much research, the fundamental mechanisms responsible for positioning chemotaxis clusters at specific sites in the membrane remain unclear [15]. Perhaps cells possess intracellular structures that anchor clusters to periodic sites along cell length [17]. However, fluorescence microscopy of cells overexpressing all chemotaxis proteins showed that the number of clusters per cell saturates well below the number of proposed cluster anchoring sites. Furthermore, the distance between chemotaxis clusters varies broadly within cells [18]. Based on those observations, Thiem and Sourjik [18] proposed that cluster nucleation and growth is a stochastic self-assembly process in which receptors freely diffuse in the membrane and then join existing clusters or nucleate new clusters. In their model, clusters nucleate anywhere in the membrane and later become attached to anchoring sites. Shortly thereafter, it was reported that anchoring sites may not be required for periodic positioning; surprisingly, simulations reveal that periodic positioning of clusters can emerge spontaneously in growing cells [19].

Direct tests of these stochastic nucleation models involve measuring, as accurately as possible, the relative spatial positioning of clusters and the distribution of cluster sizes. This requires (1) the high specificity of genetically encoded fluorescent tags and (2) spatial resolutions sufficient to count and localize single proteins, even when these proteins are densely packed. Electron microscopy has the required spatial resolution, but the density of immunogold labeling is too low to visualize a significant fraction of receptors [4]. Cryo-electron microscopy tomography has provided detailed information on large polar clusters [20],[21], but identification of individual receptors is not yet possible. Fluorescence microscopy does not have the required spatial resolution to observe individual receptors in dense clusters. Single-cell Förster resonance energy transfer (FRET) studies have been instrumental in measuring the dynamics of signaling within the chemotaxis network [12],[13], but cannot obtain the distribution of receptors inside cells.

The optical super-resolution technique photoactivated localization microscopy (PALM) combines high specificity with high resolution. In PALM, target proteins are genetically labeled with photoactivatable proteins, thus rendering them nonfluorescent until activated by near-UV light. By employing near-UV light of sufficiently low intensity, only one protein per diffraction-limited region (∼250 nm) is activated at a time. Following activation, each individual protein is then excited and imaged. Since only one protein is imaged at a time in each diffraction-limited region, the center of each molecular point spread function indicates the location of each protein [22]. Serial cycles of activation and excitation are repeated until all fusion proteins are bleached. Since individual proteins are imaged, we can count the number of proteins and computationally assemble the locations of all proteins into a composite, high-resolution image. The location of each protein can be determined to a precision of 2–25 nm, or approximately 10–100× better than the diffraction limit [23]–[26]. The localization error in each protein location depends on the number of photons collected for that protein, as well as background noise, pixel size, sample drift, and whether cells are live or chemically fixed [22],[23],[26]. The highest optical resolution is obtained with chemically fixed cells [23]. Several other optical techniques, including FPALM [27], STORM [28],[29], STED [30]–[32], and SSIM [33], also image below the diffraction limit.

Here, we use PALM images to directly test stochastic nucleation models of chemotaxis cluster self-assembly in *E. coli*. We show that many receptors are part of small clusters not previously observed in electron microscopy or fluorescence microscopy, and that these small clusters provide direct evidence for a stochastic nucleation mechanism without anchoring sites.

## Results and Discussion

### PALM Images of Chemotaxis Proteins

Three main components of the bacterial chemotaxis network (Figure 1A) were visualized by constructing photoactivatable fluorescent protein fusions to Tar, CheW, and CheY (Figure 1A, zoom). Tar is the high-abundance aspartate receptor and makes up 30%–45% of all receptors [34]. CheW is the adaptor protein, which binds all five types of chemotaxis receptors with variable stoichiometry. CheY is the chemotactic response regulator, which transduces signals from the receptors to flagellar motors. All fusion proteins were expressed from plasmids in strains lacking a genomic copy of the protein (Δ*tar* cells, Δ*cheW* cells, or Δ*cheY* cells) and are therefore nonchemotactic unless complemented (with Tar, CheW, or CheY, respectively). Labeling several distinct components of the network and comparing their localization patterns ensures that there are no confounding effects of our tags on clustering. All cells were cultured in H1, which is a defined minimal salts medium [35] that has been extensively characterized for its effects on chemotaxis protein expression [34].

#### Labeling of CheW and CheY

CheW and CheY were labeled with tandem-dimer Eos (tdEos) [24],[36], which is well characterized [24],[26],[37], bright [32], and has a contrast ratio between its on and off states sufficient to localize up to 10^5^ proteins/µm^2^ [26]. The addition of a fluorescent protein tag may affect the functionality of the original protein, therefore, we measured the functionality of CheW and CheY fusions. ΔcheW cells expressing tdEos-CheW recover their chemotaxis ability in an inducer-dependent manner; at optimal induction, cells spotted on soft-agar swarm plates with attractant swarm to 77% of the diameter of wild-type cells (Figure 1B, left; Figure S1). By contrast, ΔcheY cells expressing CheY-tdEos do not exhibit chemotaxis at any induction level, although the fusion protein does retain its ability to bind chemotaxis clusters (see below).

#### Labeling of Tar

Tar was labeled with a new photoactivatable fluorescent protein, monomer Eos (mEos) [38]. Unlike the tdEos label, the mEos label does not abolish Tar function, perhaps due to its smaller size. Δ*tar* cells expressing Tar-mEos recover their chemotaxis ability toward aspartate; at optimal induction, cells spotted on soft-agar swarm plates with aspartate swarm to 55% of the diameter of wild-type cells (Figure 1B, right; Figure S1).

Swarm plate assays of chemotaxis behavior suggest that the tdEos-CheW and Tar-mEos fusions retain some functionality, although they are not as efficient as wild-type CheW and Tar, respectively (Text S1).

#### Microscopy

**Figure 2:**
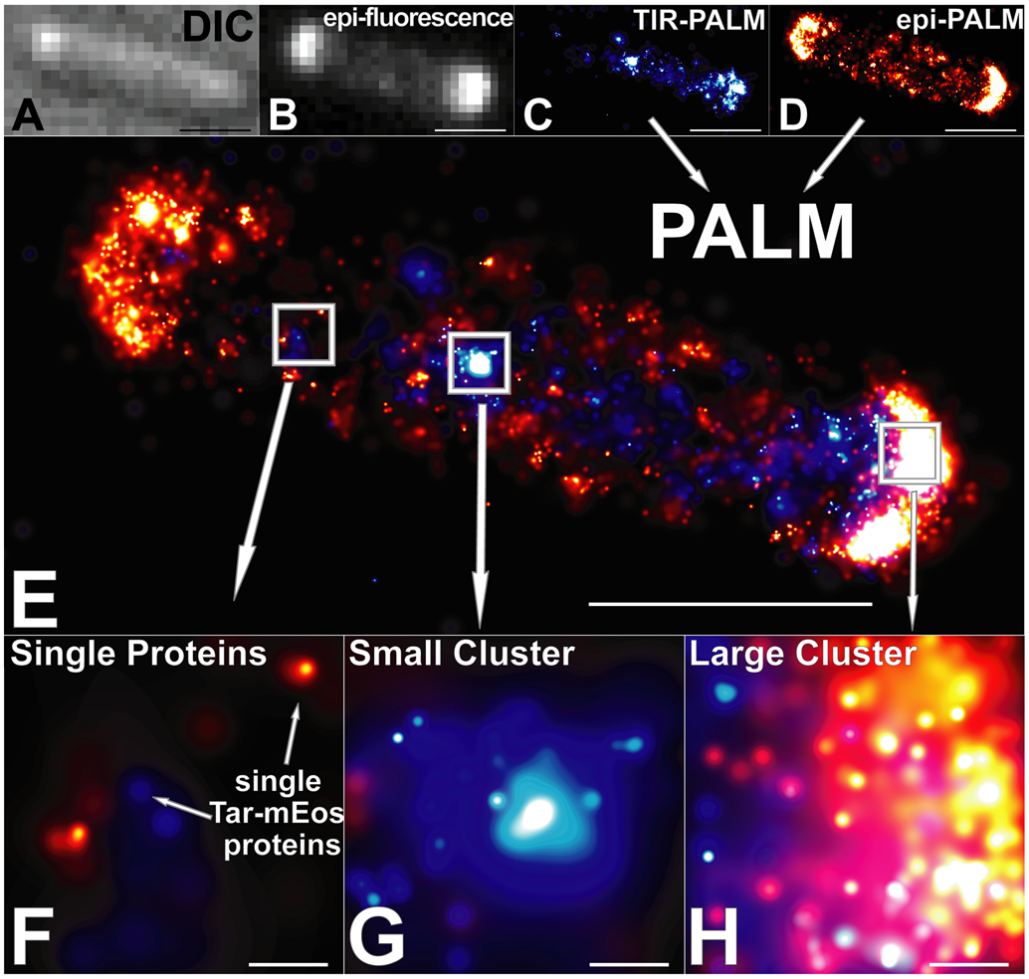
*E. coli* Δ*tar* cell with mEos-labeled Tar. (A) Differential interference contrast (DIC) image of a single cell. (B) Diffraction-limited epi-fluorescence (epi). (C) PALM image in TIR-illumination. Each protein is represented as a 2-D Gaussian distribution whose width is the positional error for that protein. (D) PALM image in epi-illumination, taken after Tar-mEos proteins in the TIR region are bleached. (E) Superposition of (C) and (D). (F) Zoom of single proteins (*n* = 44 Tar proteins) in left boxed region of (E). (G) Zoom of small cluster (*n* = 241 Tar proteins) in middle boxed region of (E). (H) Zoom of large polar cluster (*n* = 722 Tar proteins) in right boxed region in (E). Scale bar in (A–E) indicates 1 µm. Scale bar in (F–H) indicates 50 nm.

Fields of fixed *E. coli* cells were imaged in four steps. First, we visualized the cells using differential interference contrast (DIC) microscopy (Figure 2A) and diffraction-limited epifluorescence (epi) (Figure 2B). To obtain a super-resolution PALM image, we photoactivated and localized individual labeled proteins in total internal reflection (TIR) illumination (Figure 2C) until all proteins in the TIR volume (0–150 nm above the coverslip, Figure 1A) were bleached. To localize all remaining photoactivatable proteins, we used epi-illumination (Figures 1A and 2D). The use of epi-PALM in thin samples allows for imaging deeper into cells and discriminating between membrane and cytoplasmic structures. The TIR-PALM and epi-PALM data were superimposed to create a composite image (Figure 2E).

Unlike conventional microscopy (Figure 2B), PALM allowed us to see individual proteins (Figure 2F), count them (e.g., *n* = 241 Tar-mEos proteins, Figure 2G), and determine their location with a mean precision of 15±9 nm (Figure S2 and Text S1). Given this spatial precision, we cannot discern an ordered molecular arrangement of receptor dimers (Figure S3 and Text S1). Overall, we detected on average 2,770 Tar proteins, 1,340 CheW proteins, and 6,030 CheY proteins per cell (Figure S1A and S1E), consistent with native expression levels for all proteins.

Like all other fluorescence techniques, PALM does not detect every labeled protein in a cell. For example, some fluorescent proteins will not fold properly, and consequently, PALM will not detect them. The fraction of detected labeled proteins depends on induction conditions, the cell strain, and fluorescence background, all of which are similar from cell to cell in a given experiment. In this paper, we report the number of labeled proteins that are photoactivatable, emit at least 100 photons, and can be localized to better than 40 nm (See Table S1 for image parameters). Despite our underestimate of the true number of proteins, the true number of the two functional constructs (Tar and CheW) in our cells must be within two to three times the native copy numbers [20],[39]. This is because over- or underexpression of Tar or CheW impairs chemotaxis, and these complemented cells are chemotactic.

#### Image analysis

In total, we localized 1,069,281 individually labeled proteins from 326 *E. coli* cells (Figure S4). Unlike conventional microscopy, in which clusters are defined as the brightest features of an image, in PALM, the location of each individual protein is known to within approximately 15 nm, and therefore, the identification of clusters involves grouping based on interparticle separation. To objectively identify clusters, we used a tree-clustering algorithm, which groups closely spaced proteins (<30 nm; twice the mean localization precision) into clusters in agreement with those identified by eye (Figure S5). We restricted our definition of clusters to 10 or more proteins to distinguish clusters from solitary receptors.

PALM images show numerous solitary Tar receptors (Figure 2F), small clusters consisting of tens or hundreds of receptors (Figure 2G), and also the large clusters with thousands of receptors (Figure 2H) that are easily discerned in conventional fluorescence microscopy. Consistent with previous studies [4],[17],[40],[41], the largest clusters are found predominantly at cell poles.

**Figure 3:**
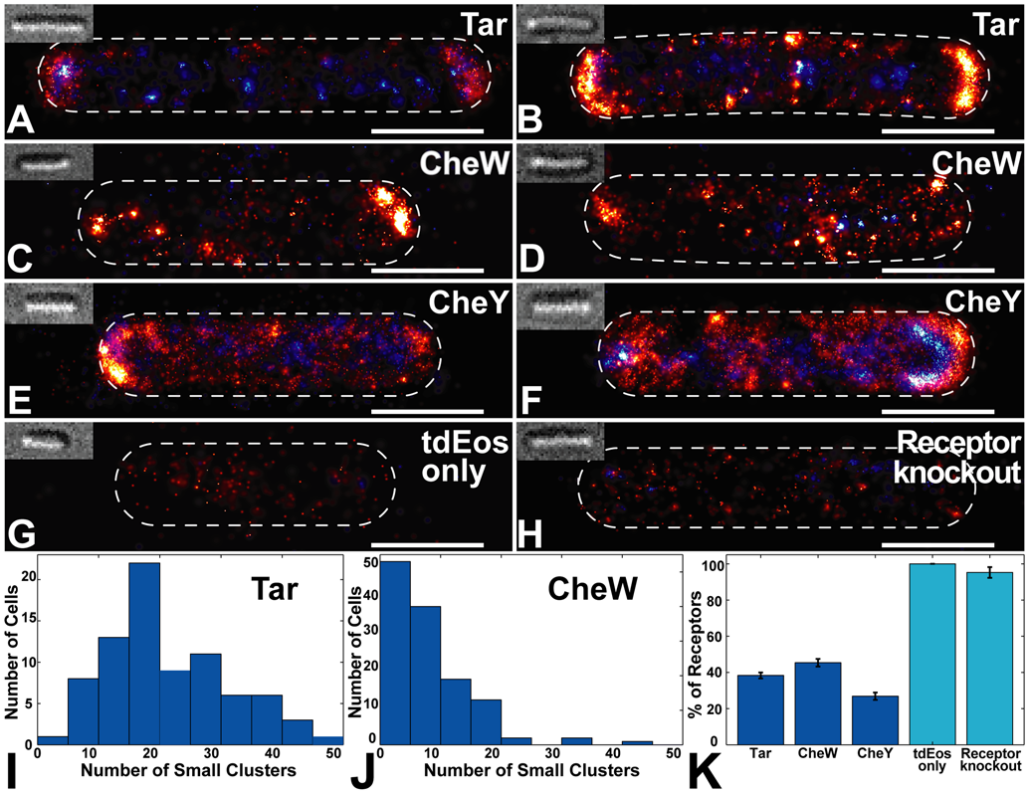
PALM images of single cells reveal small chemotaxis clusters. Single-cell PALM images containing 3,000–13,000 labeled chemotaxis proteins per cell. Largest chemotaxis clusters are found at the poles, small lateral clusters are found in all cells. DIC images (inset) correspond to cell outlines (dashed lines). (A and B) Two representative Δ*tar* cells with pALM6001 (Tar-mEos). (C and D) Two representative Δ*cheW* cells with pALM 5001 (tdEos-CheW). (E and F) Two representative Δ*cheY* cells with pALM5003 (CheY-tdEos). Although CheY-tdEos does not support chemotaxis, its abundance in polar regions suggests it retains functional interactions with chemotaxis clusters. (G) Fluorescent reporter tdEos (pALM5000) does not form clusters without fusion to chemotaxis proteins. (H) tdEos-CheW does not form clusters in a receptor knockout strain. Scale bar in (A–H) is 1 µm. (I and J) Histograms of the number of small clusters (10–100 proteins) of Tar-mEos ([I] *n* = 84 cells) or tdEos-CheW ([J] *n* = 130 cells). (K) Percentage of proteins that are found in small clusters (<100 proteins) or as solitary receptors. Error bars indicate the standard error of the mean.

Strikingly, PALM images of all three strains (Tar, CheW, and CheY) revealed small lateral clusters and solitary receptors (Figure 3A–3F) not previously observed. All cells contained a significant fraction of receptors within small clusters or as solitary receptors (Figure 3K). For example, 38% of labeled Tar receptors were found outside of large clusters (>100 proteins). Most cells (∼95%) contained between one and 48 small clusters (<100 proteins) (Figure 3I and 3J). Small lateral clusters and solitary Tar receptors were observed in all expression conditions tested. When Tar-mEos was expressed at higher levels (1 mM IPTG induction), we saw banded patterns spanning the cell length (Figure S6) that may be helical structures reflecting the organization of the general protein translocation machinery, as previously observed [16]. Many small clusters and solitary receptors were present even at this higher level of expression.

The solitary receptors are not simply imaging artifacts, since our false-positive rate is only 1–10 proteins/µm^2^; therefore, 97% to 99.5% of observed signals are correctly labeled proteins (Figure S7 and Text S1). Furthermore, the small clusters are not inclusion bodies or Eos aggregates, since tdEos alone exhibits no clustering (Figure 3G). Clustering of labeled CheW requires receptors since deletion of high-abundance chemotaxis receptors abrogated clustering of CheW (Figure 3H). Finally, a comparison between cells containing polarly localized fusion proteins (Figure 3A–3F) and control cells (Figure 3G and 3H) indicates that observed clusters are not the result of proteolysis or degradation of fusion proteins that liberate the Eos tag.

The relative spatial positioning of clusters and the precise distribution of cluster sizes contain information about the mechanism of cluster formation. For example, the exponentially distributed sizes of rain drops reflect their spontaneous aggregation and growth [42]. By contrast, the Gaussian distribution of cell lengths in *E. coli* reflects the tightly regulated processes of growth and division [43].

**Figure 4:**
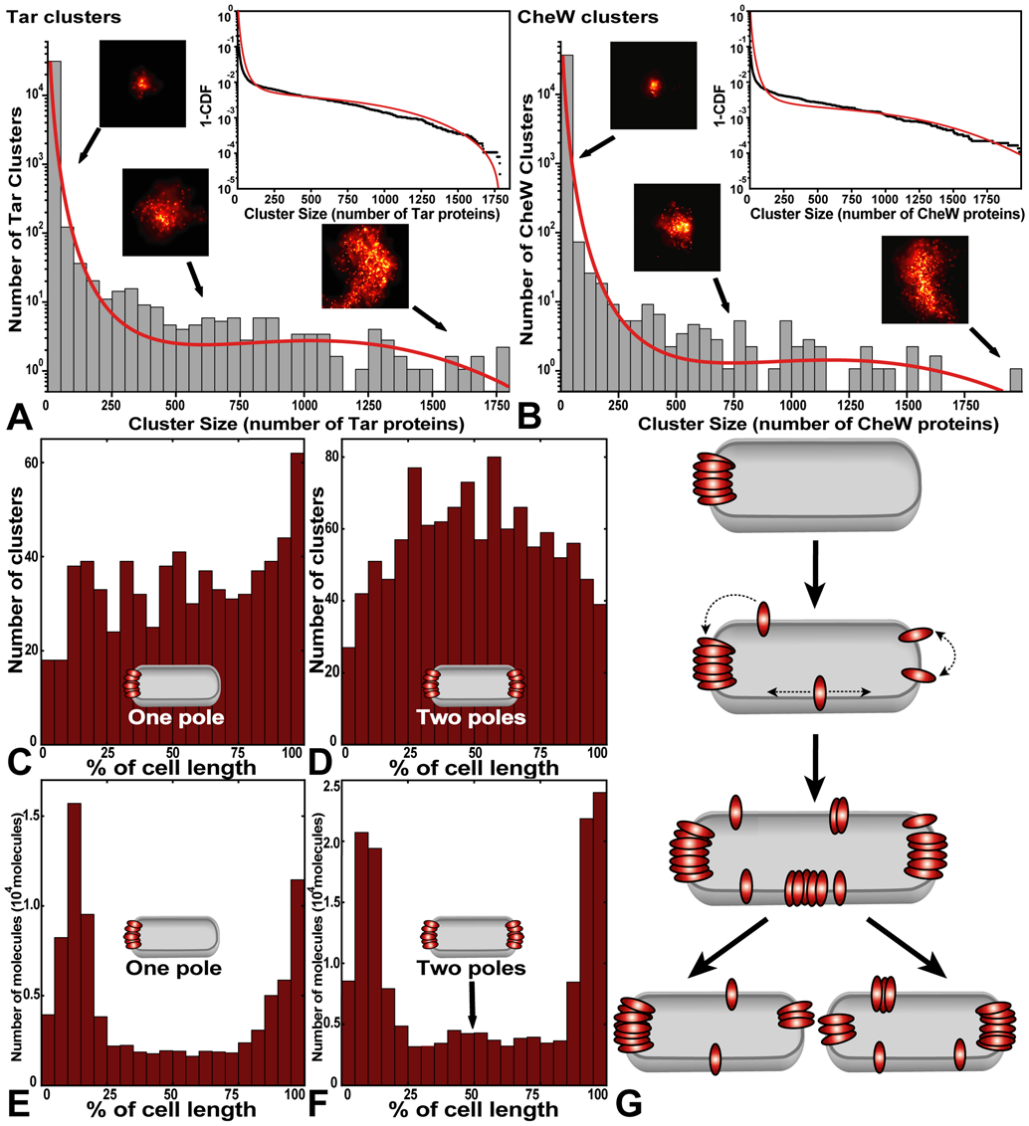
Chemotaxis cluster-size distribution and model. (A and B) Histograms of cluster size, measured by the number of closely spaced Eos-labeled Tar (A) and CheW (B) proteins. Smaller clusters occur much more frequently than larger clusters. Sample images of clusters are shown with arrows that indicate cluster size. To evaluate the fit in a bin-independent representation, we plotted the cumulative distribution function (CDF) (insets). The fit of our self-assembly model to our data is shown in red. (C and D) Cells with one (C) or two (D) large polar clusters (*n*≥400 proteins) have the highest density of remaining smaller clusters (*n*<400) furthest from the existing cluster(s). (E and F) Cells with two large polar clusters (F) exhibit higher Tar-receptor density at mid-cell (arrow) in comparison to cells with one polar cluster (E). *n* = 31 cells for (C and E), and 38 cells for (D and F). (G) Model of receptor self-assembly in which cluster locations are maintained within a population of growing and dividing cells. Cluster nucleation is most likely to occur where receptor density is high, which occurs far from any existing cluster. Dotted arrows denote receptor diffusion within the membrane.

We quantified the distribution of cluster sizes for both functional fusion proteins, Tar and CheW. Although labeled CheY appears to bind chemotaxis clusters (Figure 3E and 3F), we exclude it from further detailed analysis because it does not support functional chemotaxis; therefore, its spatial organization may not reflect native CheY. Analysis of 225,016 individual CheW proteins in 1,155 clusters and 313,937 individual Tar proteins in 2001 clusters revealed that cluster sizes were distributed according to a stretched exponential (Figure 4A and 4B), consistent with stochastic self-assembly. This distribution is not an artifact of combining data from numerous cells, since individual cells feature the same distribution (Figure S8). Since the reported protein counts underestimate the true number of labeled proteins by a constant factor, the true distribution of cluster sizes is our measured distribution scaled by a constant factor. This scaling factor does not change the stretched exponential shape of the cluster-size distribution, merely the vertical scale. We note that the total number of receptors in each cluster is on average two to three times greater than the number of Tar receptors, because Tar comprises only 30%–45% of the total number of receptors [34].

### An Extended Stochastic Nucleation Model

To understand how the distribution of cluster sizes may arise from a simple stochastic nucleation mechanism, we extended the cluster growth model of Wang et al. [19]. According to their model, receptors are inserted into the membrane at random locations and then diffuse until they are captured by a preexisting cluster or they nucleate a new cluster. The growth of a specific cluster depends on competition for receptors with nearby clusters. In our model, we treat the competing clusters as an absorbing barrier a distance *R* away from a preexisting cluster of radius *a*, which is also absorbing (Figure S9). The rate of growth of a cluster is given by 
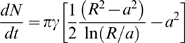
, which depends only on *R*, *a*, and γ, the deposition rate of the receptors into the membrane. Integrating 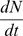 relates the size of a cluster with its age *t_age_*. In an exponentially growing population of cells, the ages of the clusters will be exponentially distributed according to 
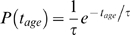
, where 1/τ is the growth rate of the cells. Assuming that receptors diffuse freely in the membrane, but clusters are stationary, we predict that the probability of a cell containing a cluster of size *N* is
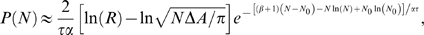
(1)where we have defined the constants α = πγ*R* ^2^ and β = 2ln(*R*)−ln(Δ*A*/π), and where *N* is the number of receptors (or receptor dimers), *N*_0_ is the number of receptors at nucleation, and Δ*A* is the area per receptor (Text S1).

In the cell membrane, small clusters would be expected to diffuse and occasionally combine with other clusters. To account for this attrition of small clusters, we modify *P*(*N*) by multiplying it by a survival probability, such that the total probability of observing a cluster of size *N* receptors is *P_tot_*(*N*) = *P*(*N*)*P_surv_*(*N*). We calculate the survival probability to be:
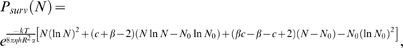
(2)where *η* is the viscosity of the membrane, *h* is the thickness of the membrane, and *c* is a constant set by the dimensions of the cell and the area per receptor (Text S1). Combining Equations 1 and 2 results in an expression with the functional form:
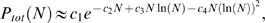
(3)which we use to fit our cluster-size distributions with free parameters *c*_2_, *c*_3_, and *c*_4_. Normalizing each cluster-size distribution fixes the constant *c*_1_.

Equation 3 fits our observed cluster-size distribution well (Figure 4A and 4B, red line), consistent with a stochastic cluster growth and nucleation mechanism. To evaluate the fit of our model to our data in a bin-independent manner, we compared the cumulative distribution function (CDF) of our cluster-size distribution with the CDF of our model (Figure 4A and 4B, insets). Importantly, our cluster growth model does not invoke cluster anchoring to cytoskeletal or predivisional structures, nor does it require active transport of receptors or clusters.

### Additional Evidence for Stochastic Nucleation

To provide further, independent, support that receptors stochastically self-assemble into clusters, we analyzed another aspect of the data. In our model, proteins that happen to be inserted close to existing clusters will be absorbed by them, whereas those inserted far from existing clusters will nucleate new clusters [19]. Thus, our model predicts that the highest density of small clusters will be found predominantly at sites that are furthest from all existing large clusters.

We identified cells with one or two large polar clusters (≥400 proteins) and measured the locations of small clusters (<400 proteins) within these cells. As predicted by our model, cells with one large polar cluster have the highest remaining cluster density at the opposite end of the cell (Figure 4C). Moreover, cells with two polar clusters have the highest cluster density in the middle of the cell, furthest from the two large clusters (Figure 4D). To ensure these results were not affected by our definition of clusters, we performed a similar analysis with receptor density. Cells with two polar clusters have significantly higher receptor density in the middle of the cells (Figure 4F, arrow) in comparison to cells with only one polar cluster (Figure 4E) (two-sample Kolmogorov-Smirnov test, *p* = 0.00013, *n* = 9,115, 19,967 proteins, middle 25% of cell length). These results are robust to changes in the specific size cutoff for large polar clusters.

Our data and modeling (Figure 4A–4F) directly support a stochastic nucleation mechanism of cluster assembly and positioning. In addition to explaining how the exponential distribution of cluster sizes arises, the model also sheds light on the mechanism for spatial self-organization along cell length, in the particular manner shown in Figure 4G and also detected by diffraction-limited imaging [17]. As cells grow, new clusters form primarily at locations that are furthest from large existing clusters. This is because the density of solitary receptors (or receptor dimers) is highest in regions furthest from existing clusters. A cell with one polar cluster will tend to form the next large cluster at the opposite pole, yielding a cell with clusters at both poles. A cell with clusters at both poles will tend to form new clusters at the cell midline, the location furthest from both poles.

In addition to generating clusters, receptor self-assembly may maintain and repair the location of clusters inside cells. In the event that a daughter cell begins without a large cluster, the first new cluster will form at a random location, but subsequent clusters nucleate furthest from that first cluster, at one of the cell poles. Furthermore, new membrane and cell wall are inserted into lateral regions of the cell [44], so that cell growth and division will eventually reposition lateral clusters at the cell poles. In this way, cells that begin without clusters will generate periodic positioning of new clusters along cell length as well as the particular exponential distribution of cluster sizes detected by PALM (Figure 4A and 4B). The mechanism of stochastic cluster formation allows cells to recover from the loss of all clusters, as well as begin to correctly position clusters soon after growth in new media. We note that our model does not address the reported difference in diffusion rates between polar and lateral clusters [17]. It is possible that the difference in membrane curvature or membrane composition between polar and lateral regions affects cluster diffusion or cluster dynamics.

There may be multiple advantages to arranging a fixed number of receptors among a variety of cluster sizes, such as fine-tuning of signal processing [45]. Our PALM images of receptors are reminiscent of the model of Berg and Purcell [46], who theorized that for optimum detection sensitivity, membrane receptors should be dispersed widely over the surface of the cell rather than concentrated in one location. In addition, recent in vitro data suggest that different densities of receptors have different kinase and methylation rates [47], suggesting that the chemotaxis network may adjust its kinase activity based on the local concentration of receptors.

Recent in vitro evidence shows that purified membrane-associated proteins can spontaneously self-assemble into complex, dynamic structures [48],[49]. Our super-resolution PALM maps of *E. coli* receptors support this notion that stochastic self-assembly can create and maintain dynamic patterns in biological membranes, without direct cytoskeletal involvement or active transport. Perhaps stochastic self-assembly is the simplest mechanism to produce robust patterns in membranes without additional machinery. Our model may apply to clustering of other proteins and to chemotaxis receptors in other organisms; however, many details are expected to be organism-specific. Analysis of super-resolution images similar to those presented here will allow counting of proteins and complexes in individual cells, reveal new levels of cell organization, and allow mechanistic hypotheses to be directly tested.

## Materials and Methods

### Bacterial Strains and Plasmids

All strains are derivatives of RP437, a chemotactic wild-type *E. coli* K-12 strain. Each chemotaxis protein was expressed in a strain lacking the genomic copy of that protein. All proteins were expressed from the inducible *trc* promoter on the medium-copy plasmid pTrc-His2 (Invitrogen) containing a pBR322-derived origin, the ampicillin resistance gene (*bla*), and the lac repressor gene (*lacI^q^*). The receptor knockout strain is HCB436 [50], which lacks all chemoreceptors except Aer and also lacks the adaptation enzymes CheR and CheB. pALM5000 contains the tandem dimer Eos (*tdEos*) gene only, and pALM6000 contains the monomer Eos (*mEos*) gene only. pALM5001, pALM5003, and pALM6001 contain *tdEos-cheW*, *cheY-tdEos*, and *tar-mEos* gene fusions, respectively.

### Photoactivatable Proteins

Eos is a photoconvertible protein that irreversibly switches its peak emission from green (516 nm) to red (581 nm) upon exposure to near-UV light [36]. Eos consists of 226 amino acids with a molecular mass of 26 kDa. Tandem dimer Eos (tdEos) consists of two copies of wild-type Eos [36] connected by a 15-residue linker SRGHGTGSTGSGSSE (nucleotide sequence TCTCGAGGTCACGGTACTGGTTCTACTGGTTCTGGTTCTTCTGAG). Monomer Eos (mEos) is the improved monomeric photostable “mEos2” from McKinney et al. [38].

### Fusion Proteins

The tandem dimer Eos (*tdEos*) gene on plasmid pALM5000 is followed by the residues ENSGS (nucleotides GAGAATTCGGGATCC) containing a BamHI site. The *tdEos-cheW* gene on plasmid pALM5001 consists of *tdEos*, a five-residue linker (ENSGS), the entire *cheW* gene (residues 1–167), and a terminal Gly-Ser encoding a BamHI site. The *cheY-tdEos* gene on plasmid pALM5003 consists of the entire *cheY* gene (residues 1–129), a one-residue linker Ala encoding part of a NcoI site, *tdEos*, and ENSGS. The monomer Eos gene (*mEos*) on plasmid pALM6000 is followed by Gly-Ser. The *tar-mEos* gene on plasmid pALM6001 consists of the entire *tar* gene (residues 1–553) joined to *mEos* with no linker, followed by Gly-Ser. The *tar* gene second codon was mutated from ATT (Ile) to GTA (Val) to introduce a NcoI site.

### Plasmid Construction

Plasmid pALM5000 was constructed by PCR amplification of the tandem dimer *Eos* gene from the plasmid ptdEos-Vinculin [26] using primers 5′-ACCATGGTGGCGATTAAGC-3′ and 5′-TTAGGATCCCGAATTCTCTCGTCTGGCATTGTC-3′ containing underlined NcoI and BamHI sites, respectively. This PCR product was inserted into plasmid pTrc-His2 (Invitrogen) according to the manufacturer's instructions. The N-terminal plasmid leader sequence was removed by digestion with NcoI and religation. pALM5001 (tdEos-CheW) was constructed by PCR amplification of *cheW* from strain RP437 using primers 5′-AAAGGTGGATCCATGACCGGTATGACGAATGTAAC-3′ and 5′-TCGGGAGGATCCCGCCACTTCTGACG-3′, and cloned into the BamHI site of pALM5000, immediately after the *tdEos* gene. pALM5003 (CheY-tdEos) was constructed by PCR amplification of *cheY* from strain RP437 using primers 5′-AGTGTGCCATGGCGGATAAAG-3′ and 5′-AGTCGCCCATGGCCATGCCCAGTTTC-3′, and cloned into the NcoI site in pALM5000, immediately before the *tdEos* gene. pALM6000 was constructed by PCR amplification of the monomeric *Eos* gene from plasmid pRSETa_mEos2 (Addgene plasmid 20341) using primers 5′-GGATCCATGGGGGCGATTAAGCCAGAC-3′ and 5′-CAAGCTTCTTAGGATCCTCGTCTGGCATTGTCAGGC-3′ containing underlined NcoI and BamHI sites, respectively. This PCR product was cloned into pALM5000, replacing *tdEos* with *mEos*. pALM6001 (Tar-mEos) was constructed by cloning a 2,345-bp synthesized DNA (DNA 2.0) into the NcoI and BamHI sites of pALM5000, replacing *tdEos* with *tar-mEos*. The synthetic DNA coded for the *tar* gene of wild-type strain MG1655 immediately followed by the monomer *Eos* gene, and the entire sequence was flanked by appropriate restriction sites. These restriction sites added a terminal Gly-Ser to the *Eos* gene.

### Strain Construction

RP437 Δ*cheW* and RP437 Δ*tar* were made by P1 transduction from the Keio collection strains JW1876 (Δ*cheW::kan*) and JW1875 (Δ*tar::kan*), respectively. The deletions in these strains were constructed to minimize polar effects on downstream gene expression by retaining the native start codon and the last 18 C-terminal nucleotides [51]. When cured of kanamycin resistance, the Keio deletion strains retain a translatable scar sequence in-frame with the deleted gene initiation codon and its C-terminal 18-nucleotide coding region. This scar sequence is expected to produce a 34-residue scar peptide with an N-terminal Met, 27 scar-specific residues, and six C-terminal gene-specific residues. RP437 Δ*cheY* was made according to Datsenko and Wanner [52], using primers that exactly removed the entire *cheY* gene and replaced it with a 1.1-kb DNA from pKD3 encoding the chloramphenicol resistance gene. Strains were cured of resistances using plasmid pCP20 as described in Cherepanov and Wackernagel [53].

### Media

Tryptone broth (T-broth) contains 1% w/v Difco Bacto-Tryptone (Becton Dickinson and Company), and 0.5% w/v NaCl (Fisher-Scientific) (pH 7.0). H1 minimal medium [35] contains 100 mM potassium phosphate (pH 7.0) (11.2 g/l K_2_HPO_4_ anhydrous, 4.8 g/l KH_2_PO_4_), 15 mM (NH_4_)_2_SO_4_, 1 mM MgSO_4_, 2 µM Fe_2_(SO_4_)_3_, with 0.5% glycerol and 1 mM required amino acids (histidine, leucine, methionine, and threonine). Minimal phosphate medium [54] contains 10 mM potassium phosphate (pH 7.0), 1 mM (NH_4_)_2_SO_4_, 1 mM MgSO_4_, 1 mM glycerol, and 0.1 mM required amino acids. Media were supplemented with 50 µg/ml ampicillin (Shelton Scientific).

### Cell Culture

Cultures were grown overnight in T-broth at 30°C with aeration. Day cultures were inoculated to an optical density at 600 nm (OD_600_) of approximately 0.01 into H1 minimal medium with appropriate antibiotics at 30°C with aeration until they reached an OD_600_ 0.1–0.3. Protein expression, when indicated, was induced by adding 10 µM IPTG for 3 h. Media and temperature were chosen to obtain the highest expression levels of properly folded proteins [36],[55].

### Swarm Plate Assay

To determine functionality of chemotaxis fusion proteins, 2 µl of stationary-phase cells were spotted on soft-agar swarm plates and incubated at 30°C for 16–18 h. Wild-type cells containing cytoplasmic Eos (positive control) were compared with appropriate deletion strains containing cytoplasmic Eos (negative control) and deletion strains with Eos-tagged chemotaxis fusions (cells used for imaging). All cells contain plasmids derived from pTrc-His2, which confers ampicillin resistance. Swarm plates contain 0.3% agar (Becton-Dickinson) in 10 mM minimal phosphate medium (or H1 medium) supplemented with 100 µM aspartate, 50 µg/ml ampicillin, and varying concentrations of IPTG. Aspartate was added to the plates to ensure that complemented mutants display chemotaxis toward aspartate, since RP437 Δ*tar* still contains the remaining four receptors that are capable of chemotaxis toward serine and oxygen. Cells were grown in tryptone broth with ampicillin at 30°C prior to spotting on swarm plates.

### Sapphire Coverslip Cleaning

Sapphire coverslips, used for their high refractive index (Olympus APO100X-CG), were placed in a 5∶1∶1 solution of Milli-Q filtered water, ammonium hydroxide, and hydrogen peroxide overnight at 75°C. The coverslips were subsequently rinsed with filtered water, sonicated in acetone for 20 min, rinsed again with water, rinsed with methanol, dried quickly under air flow, passed through a flame, and then stored dry until use.

### Sample Preparation

Clean sapphire coverslips were covered in 0.05% w/v poly-l-lysine for 30 min then rinsed with water. Cells were added and allowed to settle for 30 min at room temperature in the dark or spun onto coverslips at 2,000*g* for 10 min. Cells were fixed with 4% paraformaldehyde in 10 mM PBS (pH 7.4) for 15 min at room temperature. Fixative solution was prepared daily by mixing 0.8 g of paraformaldehyde, 18 ml of water, and 20 µl of 10 N NaOH, then dissolved by heating to approximately 50°C for several minutes with stirring, buffered to pH 7.4 with the addition of 2 ml of 10× PBS solution and 140 µl of 1 N HCl, and finally filtered. After fixation, cells were rinsed with PBS. To compensate for drift during imaging [26], a 40× dilution of 40-nm and 100-nm Au beads (Microspheres-Nanospheres, 790114-010 and 790122-010) were added.

### PALM Instrumentation

PALM imaging was performed according to Shroff et al. [24] on an Olympus IX81 inverted microscope equipped with DIC optics and a 100×, 1.65 NA objective. Laser light was delivered to the microscope through free space from a platform where 405-nm, 488-nm, and 561-nm lasers were combined. Single-molecule tdEos and mEos fluorescence signals generated during acquisition were separated from the activation and excitation light using appropriate filter sets [24] within the microscope and passed to an electron-multiplying charge-coupled device (CCD) camera running at approximately 20 Hz (50-ms exposures). Movie acquisition times were dependent on the regions of highest labeled-protein density, which are the largest chemotaxis clusters. Activation intensity was increased slowly such that a given diffraction-limited spot contained no activated proteins >90% of the time. This is necessary to ensure that only one protein is activated at a time in a single diffraction-limited spot. Image generation and data analysis were done using custom Matlab scripts (Mathworks). Acquisition times were 30–180 min for TIR, and 90–240 min for epi-illumination.

### PALM Analysis

Localization and image-rendering algorithms have been described [26]. Briefly, images were filtered and proteins were identified as signals that contained counts larger than four standard deviations above background. Proteins that became dark, but reappeared within five frames, were counted as the same protein. Only proteins that emitted at least 100 photons and had localization errors less than 40 nm were counted, and these thresholds were chosen to maximize the signal to noise for our images and minimize false positives (Figure S7). Sample drift was corrected by tracking the motion of fiducial nanoparticles, which were localized at approximately 1 Hz to better than 1 nm precision (Figure S2). Images from the TIR, epi, DIC, and brightfield channels were aligned by recording the position of fiducial nanoparticles common to all channels. All epi-PALM images were rendered with the “hot” colormap in Matlab that varies smoothly from black through shades of red, orange, and yellow to white, and TIR-PALM images were rendered with a variation of the same colormap with red and blue channels switched. Parameters used to acquire PALM data and render images are shown in Table S1.

## Supporting Information

Figure S1 Fluorescent fusion protein expression and functionality in E. coli cells.
(1.94 MB TIF)

Figure S2 Localization precision for fusion proteins including sample drift.
(1.17 MB TIF)

Figure S3 Higher localization precision is necessary to observe regular protein packing within clusters.
(2.22 MB TIF)

Figure S4 Many E. coli cells are imaged in one field of view using PALM.
(1.74 MB TIF)

Figure S5 Clustering algorithm detects clusters in agreement with those detected by eye.
(0.56 MB TIF)

Figure S6 High levels of Tar-mEos expression show banded patterns.
(4.03 MB TIF)

Figure S7 Signal and background levels for Tar-mEos and tdEos-CheW proteins.
(0.47 MB TIF)

Figure S8 All cells contain more small clusters than large clusters.
(0.31 MB TIF)

Figure S9 Model of how membrane receptor clusters grow.
(1.40 MB TIF)

Table S1 (0.08 MB DOC)

Text S1 (0.20 MB PDF)

## Abbreviations

DIC: differential interference contrast
epi: epifluorescence
PALM: photoactivated localization microscopy
TIR: total internal reflection
