# Supplementary figures and images for: Self-Organization of the Escherichia coli Chemotaxis Network Imaged with Super-Resolution Light Microscopy
Source: PLoS Biol. 2009 Jun 23;7(6):e1000137. doi: 10.1371/journal.pbio.1000137 (PMC2691949; doi:10.1371/journal.pbio.1000137)

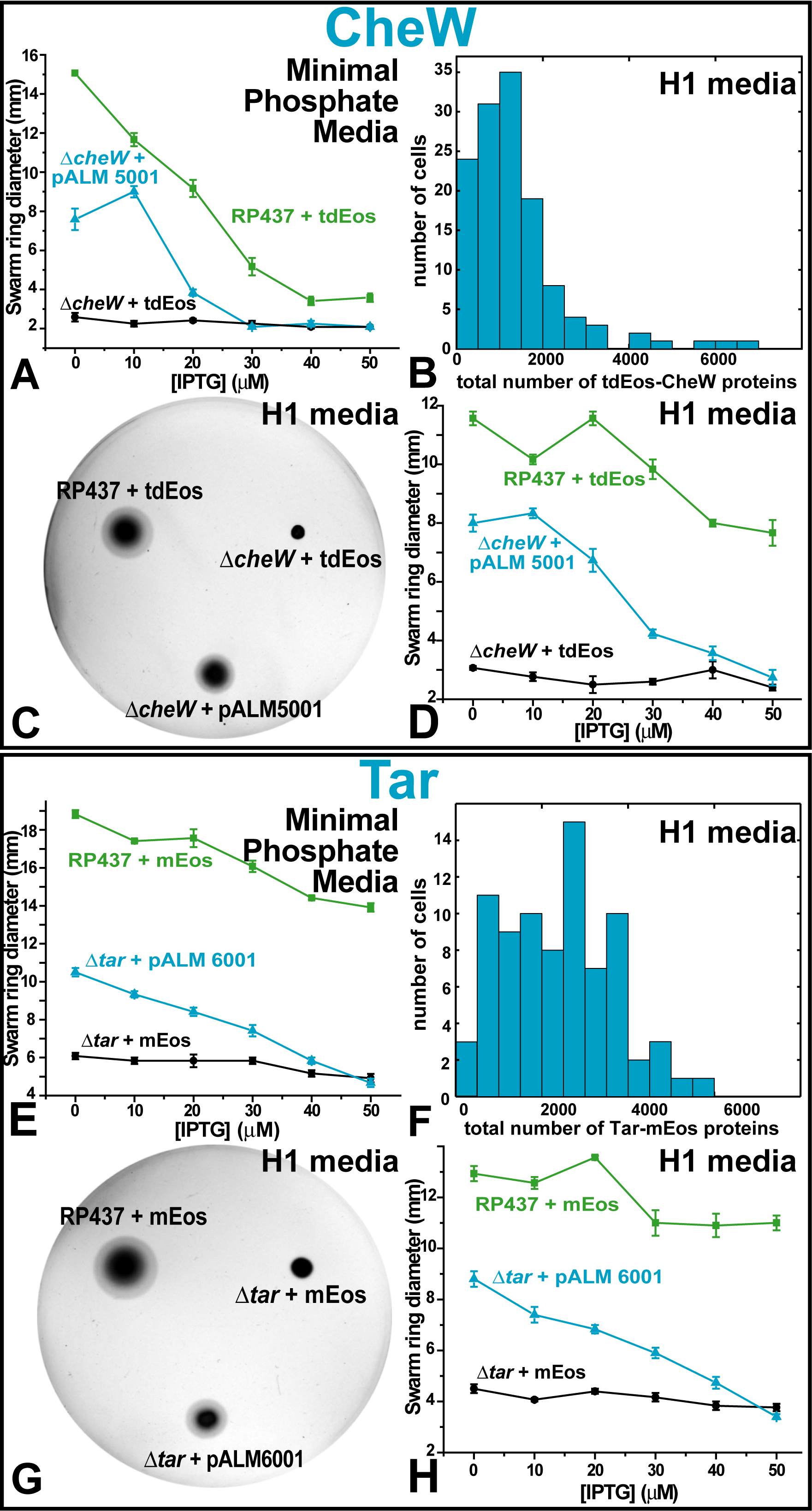

Supplement: Figure S1 — Fluorescent fusion protein expression and functionality in E. coli cells. (1.94 MB TIF) [file pbio.1000137.s001.tif]

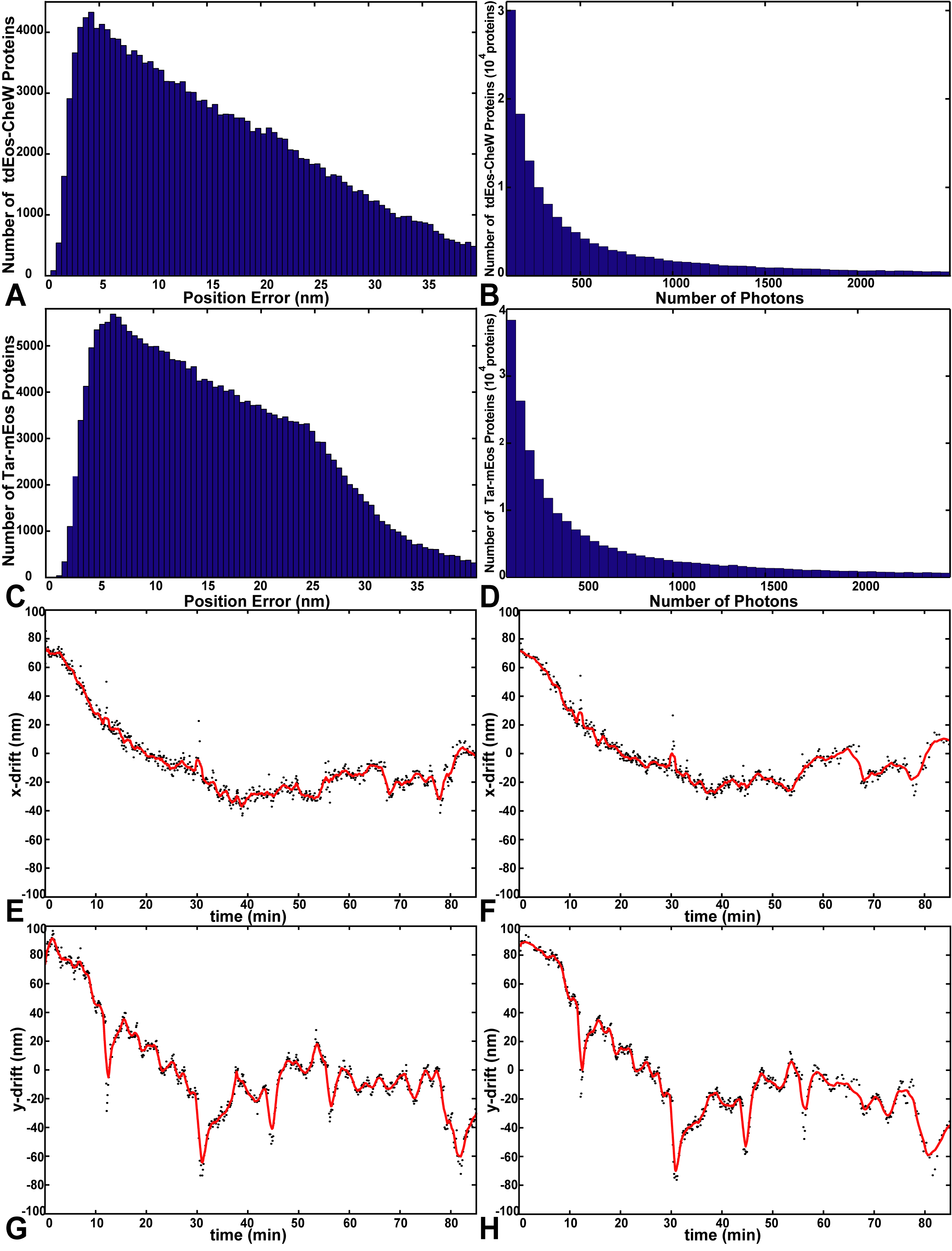

Supplement: Figure S2 — Localization precision for fusion proteins including sample drift. (1.17 MB TIF) [file pbio.1000137.s002.tif]

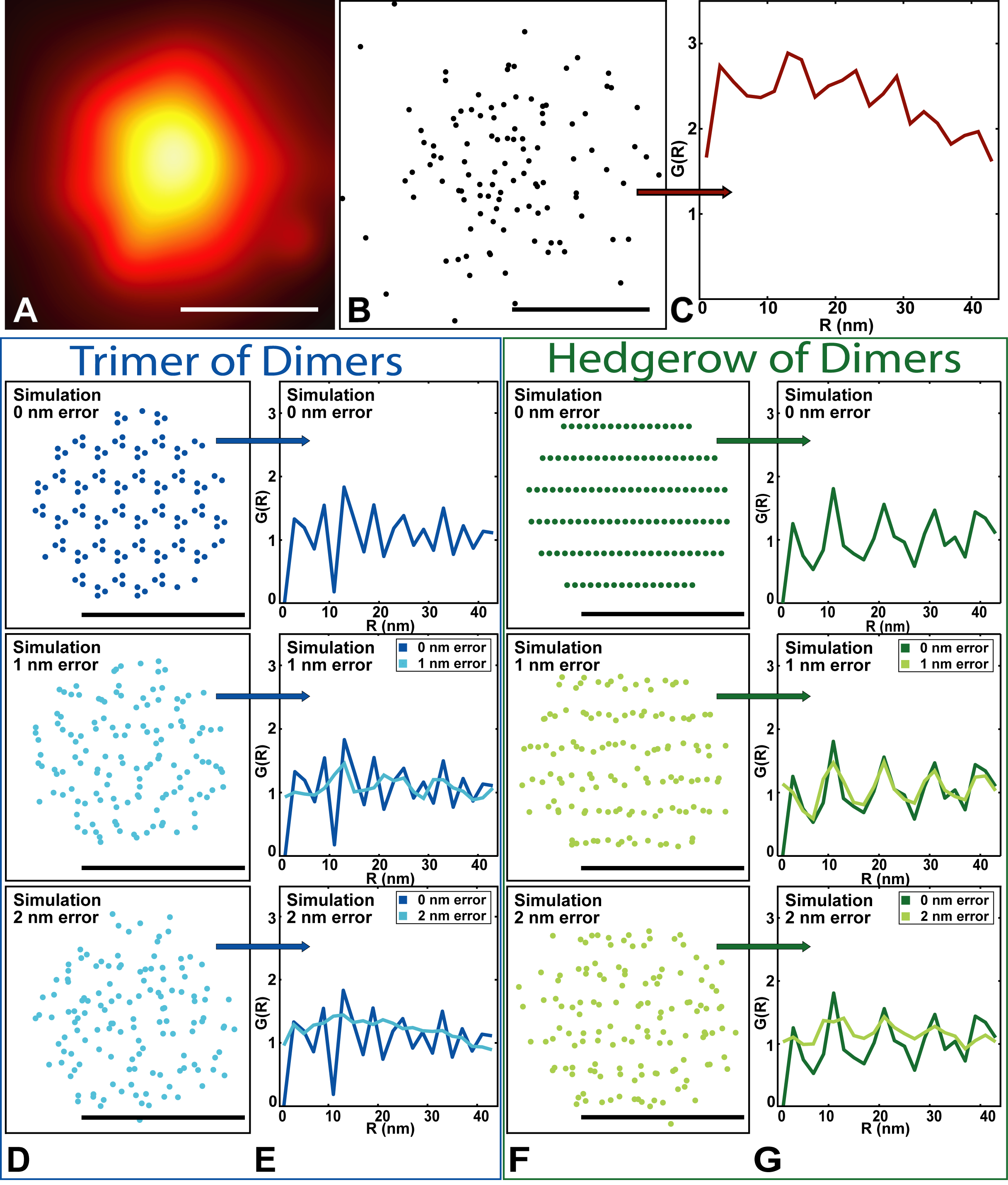

Supplement: Figure S3 — Higher localization precision is necessary to observe regular protein packing within clusters. (2.22 MB TIF) [file pbio.1000137.s003.tif]

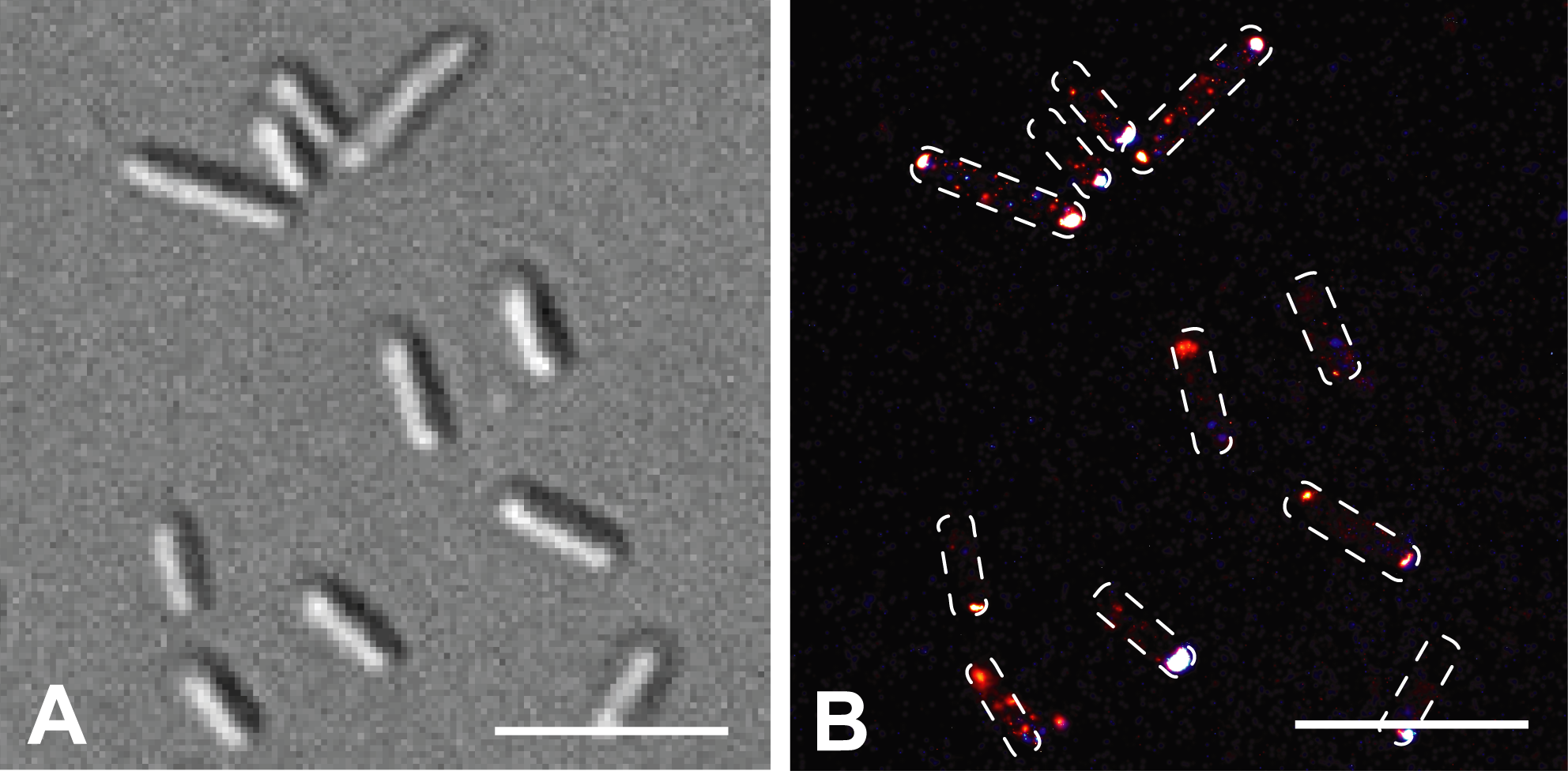

Supplement: Figure S4 — Many E. coli cells are imaged in one field of view using PALM. (1.74 MB TIF) [file pbio.1000137.s004.tif]

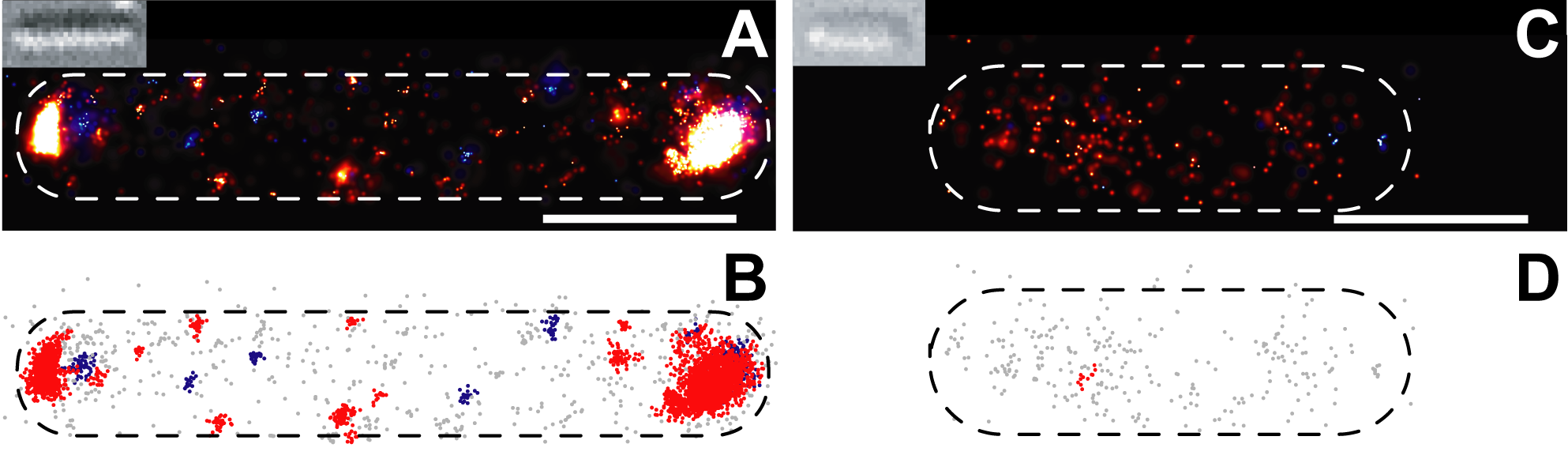

Supplement: Figure S5 — Clustering algorithm detects clusters in agreement with those detected by eye. (0.56 MB TIF) [file pbio.1000137.s005.tif]

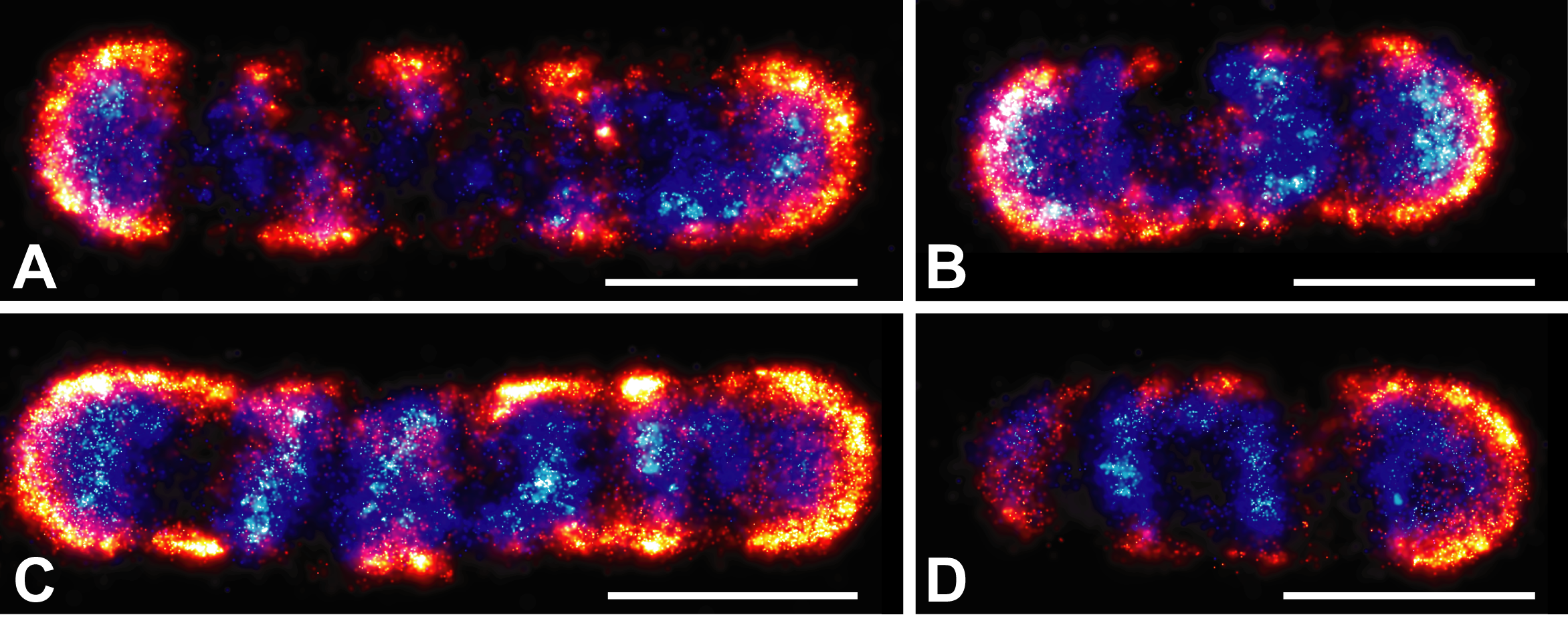

Supplement: Figure S6 — High levels of Tar-mEos expression show banded patterns. (4.03 MB TIF) [file pbio.1000137.s006.tif]

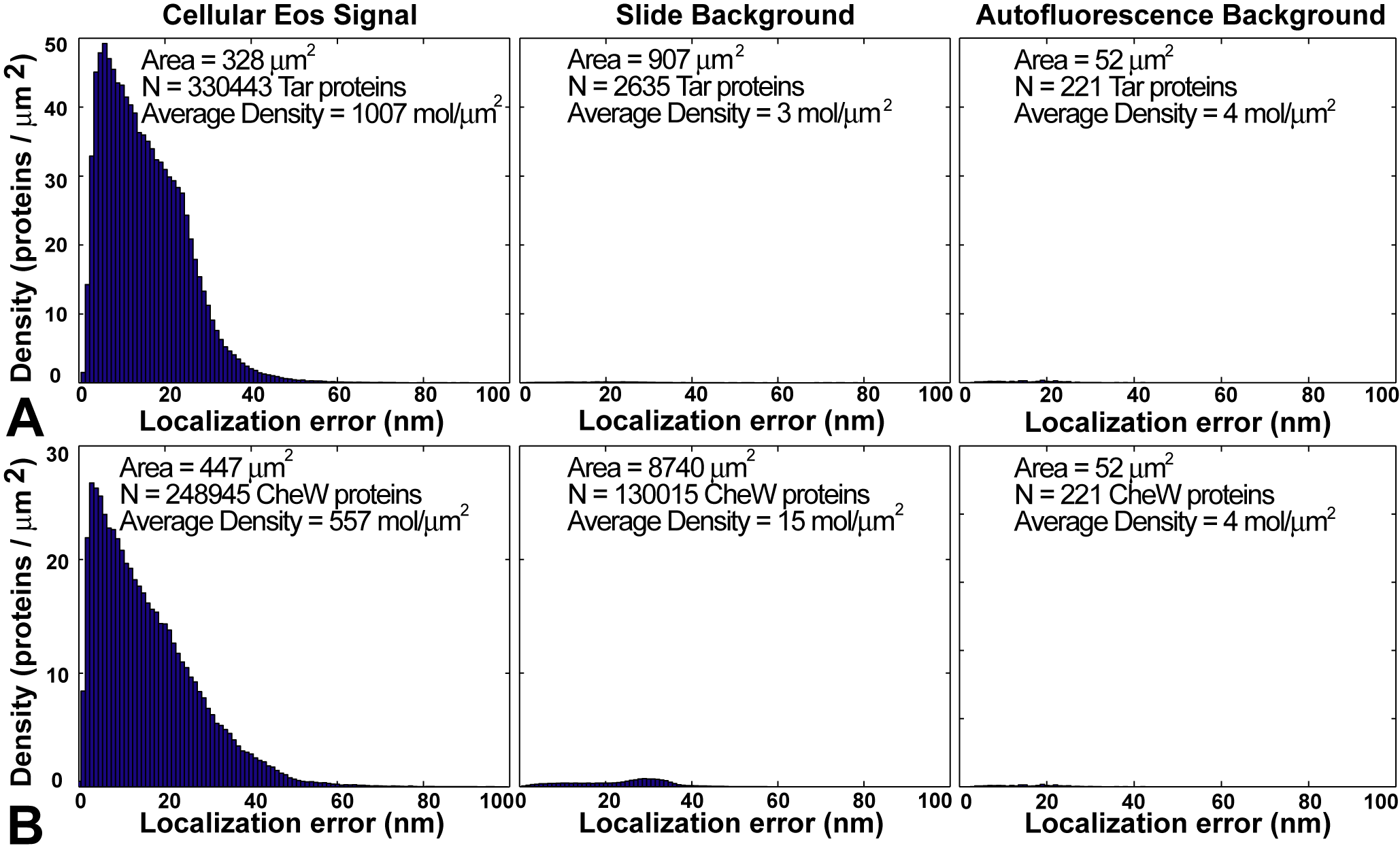

Supplement: Figure S7 — Signal and background levels for Tar-mEos and tdEos-CheW proteins. (0.47 MB TIF) [file pbio.1000137.s007.tif]

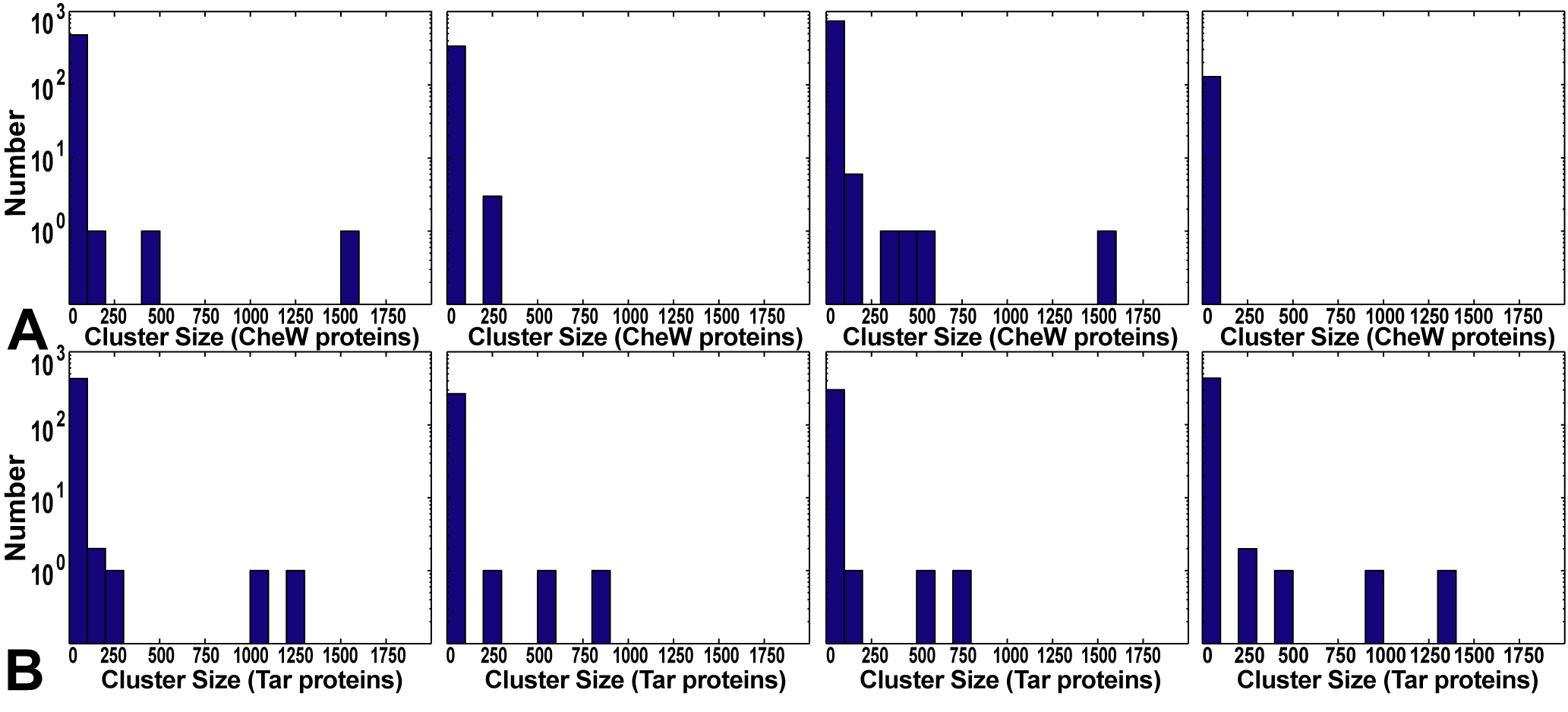

Supplement: Figure S8 — All cells contain more small clusters than large clusters. (0.31 MB TIF) [file pbio.1000137.s008.tif]

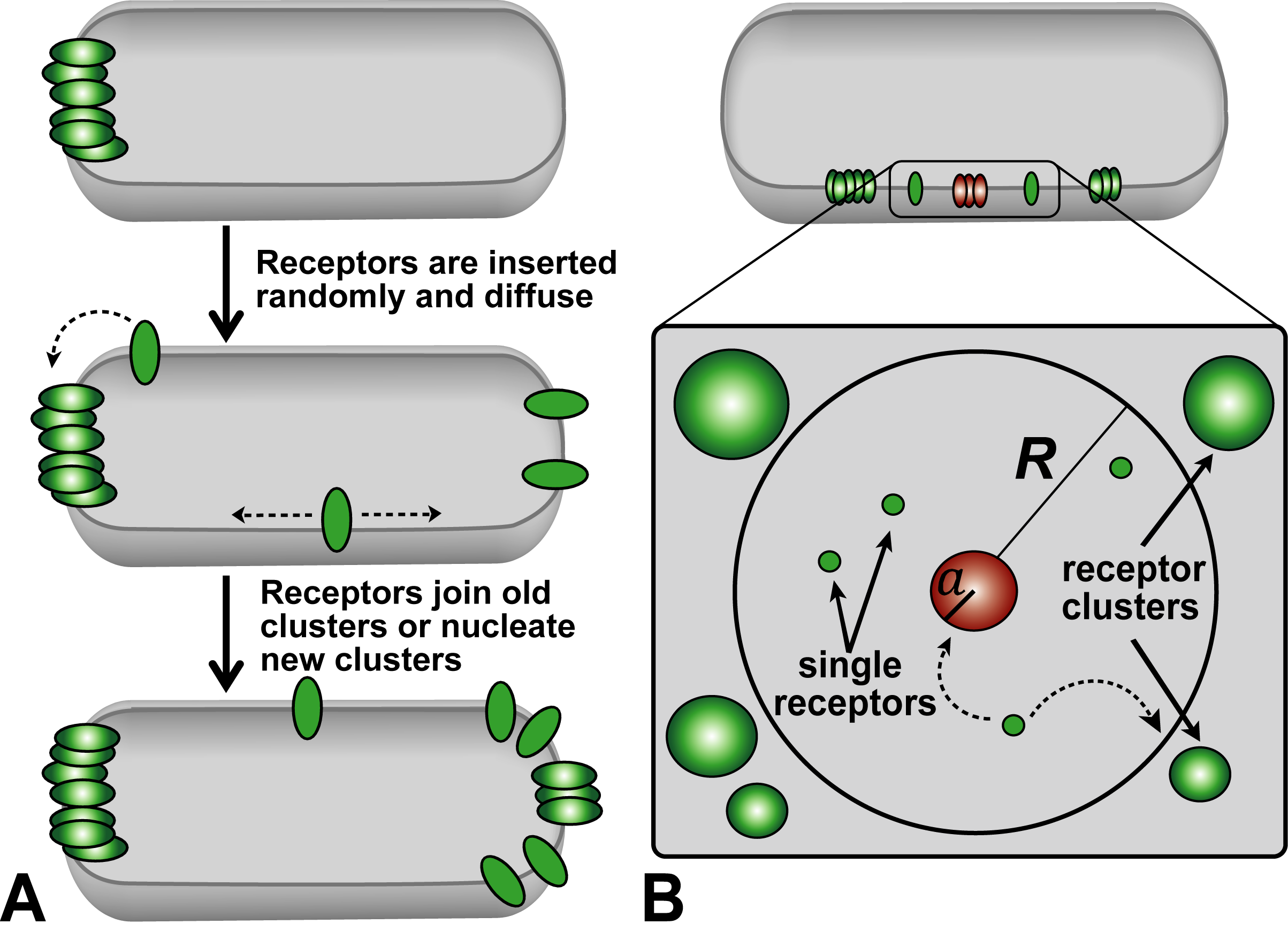

Supplement: Figure S9 — Model of how membrane receptor clusters grow. (1.40 MB TIF) [file pbio.1000137.s009.tif]
