# Supplementary material for: Self-Organization of the Escherichia coli Chemotaxis Network Imaged with Super-Resolution Light Microscopy
Source: PLoS Biol. 2009 Jun 23;7(6):e1000137. doi: 10.1371/journal.pbio.1000137 (PMC2691949; doi:10.1371/journal.pbio.1000137)
Supplement: Table S1 — (0.08 MB DOC) [file pbio.1000137.s010.doc]

**Table S1.** **Parameters of merit for the acquisition and analysis of PALM images**

|  | **F_TIR_** | **P_TIR_** | **F_epi_** | **P_epi_** | **pixelsize** | **gain** | **σ_(x,y)_** |
| --- | --- | --- | --- | --- | --- | --- | --- |
| **Figure 2C** | 83498 | 1679 | n/a | n/a | 4 | 5 x 10^4^ | 40 |
| **Figure 2D** | n/a | n/a | 83169 | 3936 | 4 | 5 x 10^4^ | 40 |
| **Figure 2E** | 83498 | 113 | 83169 | 1187 | 1 | 5 x 10^4^ | 40 |
| **Figure 2F** | 83498 | 22 | 83169 | 22 | 0.25 | 8 x 10^3^ | 40 |
| **Figure 2G** | 83498 | 217 | 83169 | 24 | 0.25 | 8 x 10^3^ | 40 |
| **Figure 2H** | 83498 | 229 | 83169 | 493 | 0.25 | 8 x 10^3^ | 40 |
| **Figure 3A** | 83498 | 1779 | 83169 | 1478 | 2.5 | 8 x 10^3^ | 40 |
| **Figure 3B** | 83498 | 1530 | 83169 | 5357 | 2.5 | 8 x 10^3^ | 40 |
| **Figure 3C** | 59600 | 271 | 79032 | 1613 | 2.5 | 2.5 x 10^4^ | 40 |
| **Figure 3D** | 64638 | 313 | 89917 | 1413 | 2.5 | 2.5 x 10^4^ | 40 |
| **Figure 3E** | 125757 | 2450 | 100369 | 5344 | 2.5 | 8 x 10^3^ | 40 |
| **Figure 3F** | 94808 | 5358 | 98755 | 7690 | 2.5 | 8 x 10^3^ | 40 |
| **Figure 3G** | 12335 | 94 | 35786 | 331 | 2.5 | 8 x 10^4^ | 40 |
| **Figure 3H** | 57986 | 289 | 102677 | 708 | 2.5 | 8 x 10^4^ | 40 |
| **Figure 4A left** | n/a | n/a | 95706 | s103 | 0.5 | 4 x 10^4^ | 40 |
| **Figure 4A mid** | n/a | n/a | 83169 | 605 | 0.5 | 3 x 10^4^ | 40 |
| **Figure 4A right** | n/a | n/a | 95706 | 1663 | 0.5 | 3 x 10^3^ | 40 |
| **Figure 4B left** | n/a | n/a | 79032 | 137 | 0.5 | 3 x 10^3^ | 40 |
| **Figure 4B mid** | n/a | n/a | 88903 | 673 | 0.5 | 2 x 10^3^ | 40 |
| **Figure 4B right** | n/a | n/a | 102388 | 1814 | 0.5 | 2 x 10^3^ | 40 |
| **Figure S3A** | 88961 | 133 | n/a | n/a | 0.3 | 1 x 10^4^ | 40 |
| **Figure S4B** | 59600 | 31493 | 79032 | 54910 | 10 | 40 | 40 |
| **Figure S5A** | 59600 | 662 | 79032 | 3982 | 2.5 | 3.2 x 10^3^ | 40 |
| **Figure S5C** | 12335 | 25 | 35786 | 264 | 2.5 | 3.2 x 10^3^ | 40 |
| **Figure S6A** | 183784 | 12881 | 122083 | 18477 | 2.5 | 5 x 10^3^ | 40 |
| **Figure S6B** | 183784 | 16836 | 122083 | 14023 | 2.5 | 5 x 10^3^ | 40 |
| **Figure S6C** | 183784 | 21087 | 122083 | 26456 | 2.5 | 5 x 10^3^ | 40 |
| **Figure S6D** | 183784 | 7890 | 122083 | 7861 | 2.5 | 5 x 10^3^ | 40 |

F_TIR_ and F_epi_ = total number of acquired frames in the image stack in TIR and epi illumination, respectively.

P_TIR_ and P_epi_ = total number of proteins localized from the data in the image stack in TIR and epi illumination, respectively.

pixelsize = size of each pixel (in nm) in the rendered image.

gain = multiplies the value in the original probability density map ([0,1] for a single protein) to obtain a value suitable for display ([0,255] for a colormap with 256 intensities). The gain is normalized for pixelsize by dividing the listed gain values by pixelsize squared before image rendering. Note that gain is comparable only across images with the same pixelsize.

σ_(x,y)_ = maximum acceptable position error (in nm) for inclusion in the final rendered image.

Note: Epi-only and TIR-only images are indicated by “n/a” in the F_TIR_ or F_epi_ fields, respectively.
