# Supplementary material for: Self-Organization of the Escherichia coli Chemotaxis Network Imaged with Super-Resolution Light Microscopy
Source: PLoS Biol. 2009 Jun 23;7(6):e1000137. doi: 10.1371/journal.pbio.1000137 (PMC2691949; doi:10.1371/journal.pbio.1000137)
Supplement: Text S1 — (0.20 MB PDF) [file pbio.1000137.s011.pdf]

Text S1

Self-organization of the *Escherichia coli* Chemotaxis Network Imaged with Super-resolution Light microscopy

Derek Greenfield[1,3,♫], Ann L. McEvoy[1,♫], Hari Shroff[4], Gavin E. Crooks[3], Ned S. Wingreen[5], Eric Betzig[4], Jan Liphardt[1,2,3*]

1 Biophysics Graduate Group and **2** Dept. of Physics, University of California, Berkeley, CA 94720, **3** Physical Biosciences Division, Lawrence Berkeley Natl. Lab, Berkeley, CA 94720, **4** Howard Hughes Medical Institute, Janelia Farm Research Campus, Ashburn, VA 20147, **5** Department of Molecular Biology, Princeton University, Princeton, New Jersey 08544, USA

[♫]These authors contributed equally to this work

Table of Contents

1. **Requirements for functional fusion proteins** 2
2. **The mean localization precision of all proteins is 15 ± 9 nm and the mean Nyquist resolution within clusters is 27 ± 8 nm** 3
3. **Our resolution is insufficient to observe regular protein packing within clusters** 4
4. **Quantification of PALM signal background (false-positive rate)** 6
5. **Derivation of the chemoreceptor stochastic self-assembly model** 7
6. **References** 12

Requirements for functional fusion proteins

The addition of a fluorescent protein tag may affect the functionality of the original protein. This is especially true when the target protein forms many functional contacts with other proteins, such as the dense arrangement of chemotaxis receptors. Therefore it is important to measure functionality for each fusion protein. For example, we observe that Tar-tdEos is non-functional as measured by chemotaxis swarm plates, yet Tar-mEos is partially functional. In this case, tdEos may be too bulky to allow functional interactions between chemotaxis receptors. In general, tags must not sterically interfere with specific surfaces of the protein, including binding sites. The tag must not aggregate or form higher ordered structures such as dimers which may affect the function or location of the protein. Tags must fold properly and should not affect the folding or stability of the protein. Finally, tags must not target the protein for degradation or modification. Predicting whether a particular fusion protein will be functional is not yet possible, therefore it is necessary to test combinations of different photoactivatable proteins fused to either the N- or C- termini of proteins, with or without a linker.

The mean localization precision of all proteins is 15 ± 9 nm and the mean Nyquist resolution within clusters is 27 ± 8 nm

When the background noise is negligible compared to the signal, the error in the fitted position for a single protein is $\sigma_{(x,y)} = s/\sqrt{N}$, where s is the standard deviation of a Gaussian approximating the true point-spread function, and N is the total number of detected photons. Since N varies for different proteins (Figures S2B and S2D), the localization error will also vary. We display PALM images with all proteins that have been localized to 40 nm or less, based on our signal-to-background analysis (Figure S7). With this threshold, the mean localization error is 15 nm with 90% of Tar proteins localized between 4 and 31 nm and 90% of CheW proteins localized between 3 and 34 nm (Figures S2A and S2C).

It is important to distinguish the localization precision of single proteins from the resolution of an image. Localization precision refers to how well the locations of individual proteins are known, whereas resolution is the ability to distinguish multiple proteins from each other. The Nyquist criterion offers a rigorous definition of resolution and specifies that, for any signal, the sampling interval must be smaller than half the desired resolution [1]. For a 2D PALM image, the Nyquist-defined spatial resolution is therefore related to the density of proteins: $\rho \geq (2/T)^2$, where T is the resolution (in nm) and ρ is the density of proteins localized to T or better (in nm^{-2}). The Nyquist resolution is highest for the densest regions of an image, which in our case, are the large polar clusters. These large clusters (> 100 fluorescent fusion proteins) each have a Nyquist resolution that varies from cluster to cluster depending on the density of labeled proteins and how well they are localized. These resolutions vary from 10 to 40 nm, with a mean resolution of 30 nm for Tar clusters and 24 nm for CheW clusters. For all large clusters, the mean Nyquist resolution is 27 nm with a standard deviation of 8 nm.

Our resolution is insufficient to observe regular protein packing within clusters

Based on crystal structures of membrane receptors [2] and cryo-electron micrographs of arrays of receptors [3,4], chemotaxis receptors are believed to assemble into tightly packed arrays of trimers of dimers [5] or hedgerows of dimers [2]. To search for repeating arrays of proteins, we first plotted the position of each protein as the center of its 2D Gaussian representation observed in the PALM image. This leads to a representation of the PALM image in which proteins are located at their most likely position (Figure S3B). We then examined these representations of dense clusters of Tar proteins and visually compared the images with two different models for how chemotaxis receptors arrange in the membrane (trimers of dimers [5] and hedgerow of dimers [2]). In each case, we did not observe any obvious arrays. We then compared radial distribution functions for clusters (example shown in Figure S3C) to the radial distribution of the ideal trimers of dimers configuration (Figure S3E top) as well as the ideal hedgerow of dimers arrangement (Figure S3G top) and found that the peaks of the measured radial distribution functions did not match the peaks of the radial distribution functions for the ideal array in either case. There was considerable variation among the Tar clusters we examined, so we compared many individual cluster radial distribution functions to the two models. We also averaged the radial distribution function from > 100 lateral clusters imaged in TIR illumination to the two models but observed no convincing alignment of peaks.

To determine whether further analysis should be performed, we estimated the resolution required to distinguish between the two models. We performed Monte Carlo simulations in which the positions of proteins in the two models were randomly moved to simulate localization error. We added random offsets to the ideal protein position array for each model by sampling errors from a scaled version of our observed error distribution (see Figure S2C). The position of the proteins as well as the radial distribution functions for simulations of σ = 1 nm and σ = 2 nm error for each model is shown (Figures S3D-G). As the error in position of the protein increases, the ideal

ordered array becomes difficult to observe. In the trimers of dimers model, $\sigma = 1$ nm of error is sufficient to obscure the array and the radial distribution function, such that neither coincides with the ideal model. The hedgerow of dimers model is distinguishable until $\sigma = 3$ nm of error in protein position, and has more obvious peaks in the radial distribution that correspond to the ideal model than in the trimers of dimers model. Our mean localization precision of 15 ± 9 nm is larger than our estimate of the required localization precision necessary to distinguish between the two models. With the invention of brighter genetically encoded fluorophores and ultra-low drift microscopes it may be possible to observe regular protein spacing in clusters, especially if clusters contain only one type of receptor.

Quantification of PALM signal background (false-positive rate)

Even a ‘bare,’ extensively cleaned coverslip will fluoresce. Additional spurious fluorescence is introduced by the cell growth medium. To quantify our general false-positive rate, we subjected the areas between cells to the same analysis we did for the cells themselves. The cell-free regions should not contain any photoactivatable proteins. This false-positive rate was 3-15 events/μm^2.

To determine the additional background from cellular autofluorescence, we counted the number of falsely-detected proteins in cells lacking fluorescent proteins. We find this background to be 4 proteins/μm^2 above the 3-15 events/μm^2.

In cells *with* photoactivatable proteins, such as Tar-mEos and tdEos-CheW, we count hundreds or thousands of proteins per μm^2. By comparing these numbers, we find that our false-positive rate is 0.3-1.5% of the average density of Tar proteins, and 0.6-3% of the average density of CheW proteins per cell (Figure S7). The background rate is this low because in PALM, non-photoactivatable background is bleached prior to image acquisition.

Derivation of the chemoreceptor stochastic self-assembly model

To understand how the distribution of cluster sizes arises within a genetically identical population of cells, we constructed a simple model. We assume that receptors are inserted into the membrane at random locations, form dimers (or trimers of dimers), and diffuse in the membrane until they are captured by a pre-existing cluster (Figure S9A). At any moment, a given cell has a particular arrangement of clusters with various sizes, and the growth of a given cluster will depend on the competition for receptors with other nearby clusters. Initially, we will assume that the clusters within a cell are stationary and do not diffuse. We would like to determine how the radius of a cluster affects the probability that a newly expressed receptor diffusing inside the membrane will be captured by that cluster.

Consider the rate of growth of a particular compact cluster of radius a. For simplicity, we treat the surrounding clusters as an absorbing boundary at radius R (Figure S9B), where R is the typical distance between clusters within the cell. Receptors (or receptor dimers) will be deposited in the annulus between a cluster of radius a and the effective absorbing boundary at radius R. Each receptor will diffuse until it is absorbed, either by the inner absorbing boundary at a or by the outer absorbing boundary at R (representing the surrounding clusters). The rate of growth of the cluster with radius a is therefore determined by the total rate at which receptors are deposited in the annulus times the fraction of receptors absorbed by the inner boundary. To solve for this fraction, consider the diffusion equation for receptors, $\frac{\partial C(r)}{\partial t} = D\nabla^2 C(r) + \gamma = 0$ (at steady-state) where $C(r)$ is the concentration of receptors at radius r within the annulus, D is the diffusion coefficient of the receptors, and γ is the insertion rate per unit area of the receptors into the membrane.

Solving the differential equation for $C(r)$ at steady state, one obtains $C(r) = -\frac{1}{4}\frac{\gamma}{D} r^2 + \frac{c_1}{D}\ln(r) + c_2$, where c_1 and c_2 are constants. After applying the

appropriate boundary conditions, $C(a) = C(R) = 0$, the solution is $C(r) = -\frac{1}{4}\frac{\gamma}{D}(r^2 - a^2) + \frac{1}{4}\frac{\gamma}{D}(R^2 - a^2)\frac{\ln(r/a)}{\ln(R/a)}$. The total current onto the circular cluster of radius a is given by $J = 2\pi r D \left.\frac{\partial C(r)}{\partial r}\right|_{r=a} = 2\pi a D\left[-\frac{1}{2}\frac{\gamma}{D}a + \frac{\gamma(R^2 - a^2)}{4Da\ln(R/a)}\right]$ which simplifies to $J = \pi\gamma\left[\frac{1}{2}\frac{(R^2 - a^2)}{\ln(R/a)} - a^2\right]$. Note that the current, J, is independent of the diffusion coefficient D but rather depends on the radius a of the cluster and the typical distance R to the surrounding clusters. These two parameters define a "basin of attraction" for a given cluster, which determines whether new receptors inserted near a given cluster will diffuse onto that cluster and be captured, or instead diffuse away to be captured by one of the surrounding clusters. When the distance between clusters is large compared to the cluster size, $R >> a$, the current is approximately $J \approx \frac{\pi\gamma}{2}\frac{R^2}{\ln(R/a)}$. If receptors are added to clusters but do not leave clusters, the growth rate of a cluster is simply the current of receptors onto that cluster,

$$\frac{dN}{dt} = J \approx \frac{\pi\gamma}{2}\frac{R^2}{\ln(R/a)}. \tag{1}$$

From the above instantaneous rate of growth of a cluster we can learn how clusters grow over time. The number N of receptors in a cluster is related to the cluster radius by $N = \pi a^2/\Delta A$, where ΔA is the area per receptor. We can therefore use $a = \sqrt{N\,\Delta A/\pi}$ to obtain the following expression for the growth rate of a cluster,

$$\frac{dN}{dt} = \frac{\pi\gamma R^2}{2\ln(R) - \ln(\Delta A/\pi) - \ln(N)} = \frac{\alpha}{\beta - \ln(N)}, \tag{2}$$

where we have defined constants $\alpha = \pi\gamma R^2$ and $\beta = 2\ln(R) - \ln(\Delta A/\pi)$. Integrating $\frac{dN}{dt}$, we obtain the expression $\int_{N_0}^{N}[\beta - \ln(N)]\,dN = \int_{t_0}^{t}\alpha\,dt$, which has the solution $\alpha(t - t_0) = (\beta + 1)(N - N_0) - N\ln(N) + N_0\ln(N_0)$. The term $(t - t_0)$ is simply the age of a cluster, $t(N)$, which we can rewrite as

$$t(N)=\frac{1}{\alpha}\left[(\beta+1)(N-N_0)-N\ln(N)+N_0\ln(N_0)\right]. \tag{3}$$

This expression relates the age of a cluster with its size; the relevant parameters are the typical distance between clusters R, the area of a receptor ΔA, the rate of insertion of new receptors into the membrane γ, and the number of receptors at nucleation N_0.

From the above relation between cluster age and cluster size, we can now estimate the distribution of cluster sizes in growing cells. In an exponentially growing population of cells, the number of cells at time t is $N_{cells}(t)\propto e^{t/\tau}$, where $1/\tau$ is the growth rate, and the total membrane surface area grows with the same exponential dependence as well. New clusters continuously nucleate such that the average number of clusters per cell is constant at steady state. Therefore, the total number of clusters also grows exponentially, such that $N_{clusters}(t)\propto e^{t/\tau}$. At a particular time t_0 the number of clusters with a given age t_{age} is the number of clusters produced at time t_0-t_{age}, or $N_{clusters}(t_0-t_{age})\propto e^{(t_0-t_{age})/\tau}$. Thus, there are more young clusters than old clusters. The probability that a given cluster is t_{age} old is $P(t_{age})\propto e^{-t_{age}/\tau}$, since $e^{t_0/\tau}$ is constant. We write this as

$$P(t)=\frac{1}{\tau}e^{-t/\tau}, \tag{4}$$

where $P(t)$ is the probability that a cluster is of age t and $1/\tau$ is the growth rate. The distribution of cluster sizes, measured by the number of proteins in a cluster, is

$$P(N)=\frac{dt}{dN}P(t(N)), \tag{5}$$

where $t(N)$ is the age of a cluster of size N receptors. Substituting Eq. 1 and 4 into Eq. 5 results in

$$P(N)\approx\frac{2}{\tau}\frac{\ln(R/a)}{\pi\gamma R^2}e^{-t(N)/\tau}. \tag{6}$$

Finally, substituting Eq. 3 into Eq. 6 results in an expression for the distribution of cluster sizes as a function of N, the number of receptors in a cluster,

$$P(N)\approx\frac{2}{\tau\alpha}\left[\ln(R)-\ln\sqrt{N\Delta A/\pi}\,\right]e^{-[(\beta+1)(N-N_0)-N\ln(N)+N_0\ln(N_0)]/\alpha\tau}, \tag{7}$$

where the approximation holds when the distance between clusters is large compared to the size of a cluster, and for clusters large enough to not diffuse appreciably.

However, small clusters in particular would be expected to diffuse within the cell membrane, leading to the attrition of some clusters of size N as they aggregate with other clusters. To account for this loss of clusters by diffusion and aggregation, Eq. 6 is modified by a survival probability, P_{surv}, such that

$$P_{tot}(N) = P(N)\, P_{surv}(N)\,. \tag{8}$$

If a cluster of size N has an attrition rate of $\mu(N)$, then

$$P_{surv} = e^{-\int_0^{t(N)} \mu\left(N(t')\right)dt'} = e^{-\int_{N_0}^{N} \mu(N')\left(\frac{dN'}{dt'}\right)^{-1} dN'}\,. \tag{9}$$

Substituting the expression for dN/dt (Eq. 2) into Eq. 9, we obtain

$$P_{surv}(N) = e^{-\int_{N_0}^{N} [\mu(N')(\beta - \ln N')/\alpha]\, dN'}\,. \tag{10}$$

To determine the attrition rate $\mu(N)$, we assume that the rate of attrition via cluster diffusion and aggregation is the inverse of the typical time for a cluster of size N to diffuse to the boundary at R, so that $\mu(N) = D(N)/R^2$, where $D(N)$ is the diffusion coefficient for a cluster of size N. For diffusion in a two-dimensional membrane, $D = kTb$, where k is the Boltzmann constant, T is temperature, the motility is $b = \frac{1}{4\pi\eta h}(l_c - \ln(a))$ (see [6]), η is the viscosity of the membrane, h is the thickness of the membrane, a is the cluster radius, and l_c is a cutoff set by the dimensions of the cell. Therefore,

$$\mu(N) = \frac{D(N)}{R^2} = \frac{kT}{4\pi\eta h R^2}\left(l_c - \ln\sqrt{N\Delta A/\pi}\right). \tag{11}$$

Substituting Eq. 11 into Eq. 10,

$$P_{surv}(N) = e^{-\int_{N_0}^{N} \frac{kT}{8\pi\eta h R^2}(c - \ln N')[(\beta - \ln N')/\alpha]\, dN'}\,, \tag{12}$$

where $c = 2l_c - \ln(\Delta A/\pi)$. Performing the integral in the exponent yields

$$P_{surv}(N) = e^{\frac{-kT}{8\pi\eta h R^2 \alpha}\left[N(\ln N)^2 + (c+\beta-2)(N\ln N - N_0 \ln N_0) + (\beta c - \beta - c + 2)(N - N_0) - N_0(\ln N_0)^2\right]}\,. \tag{13}$$

Recall that the probability, $P_{tot}(N)$, of observing a cluster of size N is given by the product of $P(N)$ and $P_{surv}(N)$. While some of the parameters in $P_{tot}(N)$ are not known, from the above analysis the functional dependence of $P_{tot}(N)$ on N is known to be

$$P_{tot}(N) \approx c_1 e^{-c_2 N + c_3 N \ln(N) - c_4 N (\ln(N))^2}, \tag{14}$$

where we neglect a weak (logarithmic) N dependence of c_1. We can use Eq. 14 to fit our histograms of cluster sizes. Normalizing the distribution fixes the constant c_1. We fit $P_{tot}(N)$ to our normalized distribution using unconstrained nonlinear optimization with the free parameters c_2, c_3, and c_4.

The good fit of our model (Figures 4A and 4B) to the data strongly suggests that cluster growth is the result of simple receptor aggregation, not complex biological regulation. Importantly, the distribution of sizes does not result from an equilibrium partitioning of receptors among clusters, but rather from the continuous growth and aggregation of clusters in an exponentially growing and dividing population of bacteria.

References

1. Shannon CE (1949) Communication in the presence of noise. Proceedings of the Institute of Radio Engineers 37: 10-21.
2. Park S, Borbat P, Gonzalez-Bonet G, Bhatnage J, Pollard A, et al. (2006) Reconstruction of the chemotaxis receptor-kinase assembly. Nature Structural & Molecular Biology 13: 400-407.
3. Weis R, Hirai T, Chalah A, Kessel M, Peters P, et al. (2003) Electron microscopic analysis of membrane assemblies formed by the bacterial chemotaxis receptor Tsr. Journal of Bacteriology 185: 3636-3643.
4. Kim KK, Yokota H, Kim S-H (1999) Four-helical-bundle structure of the cytoplasmic domain of a serine chemotaxis receptor. Nature 400: 787-792.
5. Briegel A, Ding HJ, Li Z, Werner J, Gitai Z, et al. (2008) Location and architecture of the *Caulobacter crescentus* chemoreceptor array. Molecular Microbiology 69: 30-41.
6. Saffman PG, Delbrück M (1975) Brownian motion in biological membranes. Proceedings of the National Academy of Sciences of the United States of America 72: 3111-3113.
